# Enhancing radiosensitivity of osteosarcoma by ITGB3 knockdown: a mechanism linked to enhanced osteogenic differentiation status through JNK/c-JUN/RUNX2 pathway activation

**DOI:** 10.1186/s13046-025-03417-4

**Published:** 2025-05-24

**Authors:** Qiujian Lian, Hu Liu, Jingyan Li, Cheng Luo, Chang Liu, Haonan Zhao, Peijun Dai, Bingxuan Wang, Huipeng Zhou, Xin Jiang, Zhiwei Wang, Suchi Qiao

**Affiliations:** 1 Department of Orthopedics, Fuzhou Second General Hospital, Fuzhou, Fujian 350007 China; 2 Department of Orthopedics, The Third Affiliated Hospital of Naval Medical University, Shanghai, 201805 China; 3 Radiation Medicine, Faculty of Naval Medicine, Naval Medical University, Shanghai, 200433 China; 4 Department of Geriatric Medicine, Fujian Provincial Hospital, Fuzhou, Fujian 350001 China; 5 Department of Orthopedics, The 900th Hospital of Joint Logistic Support Force, Fuzhou, Fujian 350025 China; 6 Department of Anesthesiology, The Second Affiliated Hospital of Naval Medical University, Shanghai, 200003 China

**Keywords:** ITGB3, Radiosensitizing, Osteosarcoma, JNK, RUNX2, Osteogenic differentiation

## Abstract

**Background:**

The prognosis of osteosarcoma has improved little over the past few decades, with radioresistance being a contributing factor. Effective radiosensitizing targets and novel mechanisms for treating osteosarcoma are urgently needed. Research on the impact of regulating differentiation levels on the radiosensitivity of malignant tumors is limited. This study aimed to explore the efficacy of ITGB3 as a novel radiosensitizing target in osteosarcoma and to explore whether the modulation of osteogenic differentiation plays a role in mediating the radiosensitizing effect.

**Methods:**

RNA sequencing was utilized to screen for potential targets that affect the radiosensitivity of osteosarcoma. In vitro assays examining cell viability, apoptosis, proliferation, migration, and invasion were conducted to verify the radiosensitizing effect of ITGB3-knockdown (KD). Furthermore, in vivo validation was performed by constructing mouse models with subcutaneous and orthotopic tibial tumors. Rescue experiments involving siRNAs and molecular inhibitors were performed to explore and validate the mechanisms through which ITGB3-KD exerts a radiosensitizing effect in vitro and in vivo. Additionally, osteogenic differentiation cultures of osteosarcoma cells were conducted as auxiliary validation for the radiosensitizing mechanism.

**Results:**

ITGB3-KD had a radiosensitizing effect on osteosarcoma in vitro by inhibiting cell viability, proliferation, migration, and invasion and promoting apoptosis. ITGB3-KD radiosensitized osteosarcoma in vivo in subcutaneous and orthotopic tibial tumor models. ITGB3-KD upregulated the JNK/c-JUN pathway, and rescue experiments with a JNK inhibitor revealed that the activation of this pathway was crucial for the upregulation of osteogenic markers such as RUNX2, OCN, and OPN, as well as for promoting apoptotic pathways. siRNA-based rescue experiments indicated that the upregulation of RUNX2 mediated the proapoptotic radiosensitizing effects of ITGB3-KD. Culture in osteogenic differentiation medium promoted osteosarcoma radiosensitization by enhancing the osteogenic differentiation status, working synergistically with ITGB3-KD.

**Conclusions:**

Our findings indicate that ITGB3-KD enhances radiosensitivity in osteosarcoma by promoting osteogenic differentiation and apoptosis through activation of the JNK/c-JUN/RUNX2 pathway, identifying ITGB3 as a candidate therapeutic target and implicating JNK/c-JUN/RUNX2 signaling as a modulatory axis for improving the response to radiation of osteosarcoma.

**Supplementary Information:**

The online version contains supplementary material available at 10.1186/s13046-025-03417-4.

## Introduction

Osteosarcoma is recognized as a highly malignant bone tumor that arises from multipotent mesenchymal precursor cells [1]. Given its histological derivation, cancer stem cell subpopulations, and clinical pathological evidence of tumor bone, osteosarcoma possesses osteogenic differentiation potential [1–4].

The management of osteosarcoma has remained largely unchanged since the 1980s, relying on a standard triad of neoadjuvant chemotherapy, surgical resection, and adjuvant chemotherapy—a regimen that initially revolutionized patient outcomes. However, therapeutic progress has stagnated over the past decades, with the 5-year overall survival rate plateauing at approximately 70% for patients with localized disease [5, 6]. Notably, striking disparities persist: metastatic and chemotherapy-resistant patients exhibit 5-year event-free survival rates below 20%, even with aggressive treatment protocols [6, 7]. This clinical landscape underscores the need to explore novel strategies, including targeted therapies, immunotherapies, and radiosensitization approaches.

Radiotherapy, although pivotal for radiosensitive malignancies, has limited application in osteosarcoma because of its inherent radioresistance [8]. Radiosensitizers could increase osteosarcoma radiosensitivity, thereby enabling a reduction in target doses and facilitating diverse applications, including preoperative tumor reduction, adjuvant treatment for positive surgical margins, alternative therapy for nonsurgical candidates, and salvage therapy in cases of recurrence. Consequently, radiotherapy could emerge as an important supplemental modality for treating osteosarcoma.

Malignant tumors harbor a subpopulation of cancer stem cells (CSCs) characterized by their high cellular differentiation potential and notably low radiosensitivity [9]. Recent research has focused on eliminating CSCs or stimulating their differentiation as strategies to mitigate the radioresistance of malignant tumors [10–14]. Additionally, a suboptimal osteogenic differentiation status has been implicated as a contributing factor to cisplatin resistance in osteosarcoma [15, 16]. Collectively, these findings suggest the potential influence of modulating the differentiation status of malignant tumor cells on both radiosensitivity and chemosensitivity.

Despite the inherent osteogenic differentiation potential of osteosarcoma [1–4], the low expression levels of osteogenic markers, such as RUNX2 (RUNX family transcription factor 2), OCN (osteocalcin), and OPN (osteopontin), observed in numerous human osteosarcoma cell lines suggest that osteogenic differentiation defects are a prevalent characteristic of osteosarcoma [17, 18]. This finding partly elucidates its resistance to both radiotherapy and chemotherapy. To our knowledge, no studies have explored the impact of regulating osteogenic differentiation on the radiosensitivity of malignant tumors, including osteosarcoma.

ITGB3 (integrin subunit beta 3) forms the integrins α2bβ3 (with ITGA2B) and αvβ3 (with ITGAV), which play important physiological roles as transmembrane glycoproteins [19, 20]. The overexpression of ITGB3 inhibits mineralization in osteogenic precursor cells [21], whereas its downregulation promotes phenotypic transformation of osteoclasts and calcification of arterial vessel walls [22]. Furthermore, the downregulation of ITGB3 improves osteoporosis by inhibiting osteoclast viability and bone resorption capacity [23]. These findings suggest the complex involvement of ITGB3 in bone formation and resorption processes. Additionally, ITGB3 expression has been associated with increased cisplatin resistance in osteosarcoma [24]. Given the previously mentioned connection between differentiation status and radiosensitivity/chemosensitivity, we hypothesize that increased ITGB3 expression may contribute to the resistance of osteosarcoma to radiotherapy and chemotherapy by inhibiting osteogenic differentiation. However, further testing is needed to validate this hypothesis thoroughly. Previous reports using pan-integrin inhibitors have provided preliminary evidence implicating αvβ3 and αvβ5 integrins in tumor radiosensitization [25, 26]. However, the specific contribution of ITGB3 (β3 integrin subunit) to this process remains underexplored, particularly in the context of subunit-selective targeting strategies.

In the present study, ITGB3 was initially identified as a target that modulates the radiosensitivity of osteosarcoma through RNA sequencing (RNA-seq). The radiosensitizing effect of ITGB3-knockdown (KD) was subsequently validated in both in vitro and in vivo osteosarcoma models. We focused our mechanistic investigations on osteogenic differentiation pathways. We demonstrated that the knockdown of ITGB3 increased radiosensitization by promoting osteogenic differentiation and apoptosis through activation of the JNK/c-JUN/RUNX2 pathway in osteosarcoma.

## Results

### ITGB3, upregulated by irradiation in osteosarcoma cells, is a potential radiosensitization target

**Fig. 1:**
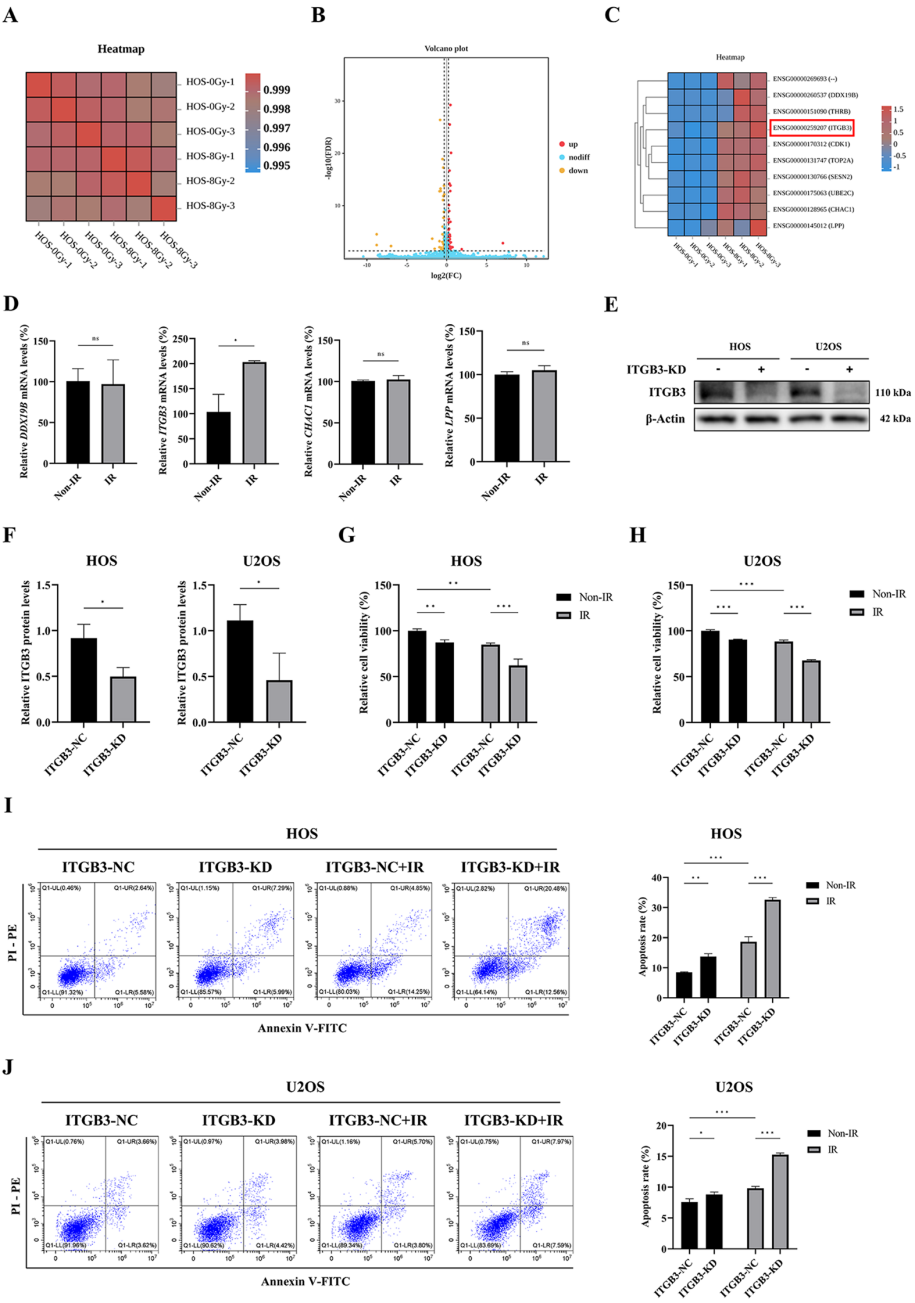
ITGB3 is identified primarily as a radiosensitizing target in osteosarcoma via RNA-seq and subsequent validation. HOS cells treated with 8 Gy of irradiation (IR) or non-IR were collected 12 h posttreatment for RNA-seq (*n* = 3 per group). Correlation heatmap (**A**) displaying the correlation between samples. The overall distribution of differentially expressed genes (**B**) was further refined to showcase the top 10 upregulated gene lists post-IR (**C**). Using qPCR, the mRNA expression levels (**D**) of DDX19B, CHAC1, LPP, and ITGB3 were quantified in HOS cells collected 12 h after being subjected to either 8 Gy of IR or non-IR treatment (*n* = 3 per group). Short-term ITGB3-knockdown (ITGB3-KD) and ITGB3-negative control (ITGB3-NC) HOS and U2OS cells were constructed using siRNAs, and the knockdown effect of ITGB3 protein (**E**, **F**) was demonstrated by western blot results 24 h after siRNA transfection. The HOS (**G**) and U2OS (**H**) cells were subjected to 8 Gy of IR or non-IR treatment 24 h after siRNA transfection, and the viability of cells 24 h posttreatment was detected using by Cell Counting Kit-8 assay (*n* = 6 per group). The HOS (**I**) and U2OS (**J**) cells were subjected to 8 Gy of IR or non-IR treatment 24 h after siRNA transfection, and the apoptosis rates 24 h posttreatment were detected using flow cytometry (*n* = 3 per group). *P<0.05, **P<0.01, ***P<0.001, ns = not significant

To elucidate gene responses in osteosarcoma after irradiation (IR) and explore potential gene targets affecting radiosensitivity, total RNA was extracted 12 h postintervention from HOS human osteosarcoma cells treated with 8 Gy of IR or non-IR conditions for RNA-seq analysis. The sample correlation heatmap demonstrated excellent intragroup reproducibility (Fig. 1A). Differential gene expression analysis between groups revealed 66 upregulated and 45 downregulated genes in the IR-treated group (Fig. 1B). Considering that upregulated genes post-IR often mediate radioresistance, the top 10 upregulated genes (based on log2-transformed fold change values) in the IR-treated group were prioritized for further validation (Fig. 1C). Genes previously reported to affect radiosensitivity in malignancies, such as *THRB*,* CDK1*,* UBE2C*,* SESN2*, and *TOP2A*, along with *ENSG00000269693*, due to the lack of commercial antibodies, were excluded from further analysis. Instead, the unexplored genes *ITGB3*,* DDX19B*,* LPP*, and *CHAC1* were validated for expression using real-time quantitative PCR (qPCR) under identical intervention conditions and sampling time points as those used for the RNA-seq analysis. This confirmed that *ITGB3* mRNA exhibited a similar upregulation trend post-IR, which was consistent with the RNA-seq data (Fig. 1D). Given the absence of similar trends in other candidate genes, ITGB3 was prioritized for subsequent investigation.

Based on siRNA transfection, short-term ITGB3-knockdown (ITGB3-KD) and ITGB3-negative control (ITGB3-NC) HOS and U2OS osteosarcoma cells were constructed, and the effectiveness of ITGB3 protein knockdown was verified by western blotting (Fig. 1E, F). A Cell Counting Kit-8 (CCK-8) assay demonstrated that ITGB3-KD alone inhibited the viability of both HOS and U2OS cells, whereas the combination of ITGB3-KD and IR notably suppressed cell viability further, with statistical analysis suggesting a synergistic interaction between the two treatments (Fig. 1G, H). Furthermore, the results of the apoptosis assay indicated that ITGB3-KD alone induced a weaker apoptotic effect in HOS and U2OS cells than did IR. However, the combination of ITGB3-KD and IR markedly promoted apoptosis, with statistical analysis indicating a synergistic effect between the two factors (Fig. 1I, J).

### The combination of ITGB3 knockdown and irradiation has a radiosensitizing effect on osteosarcoma in vitro

**Fig. 2:**
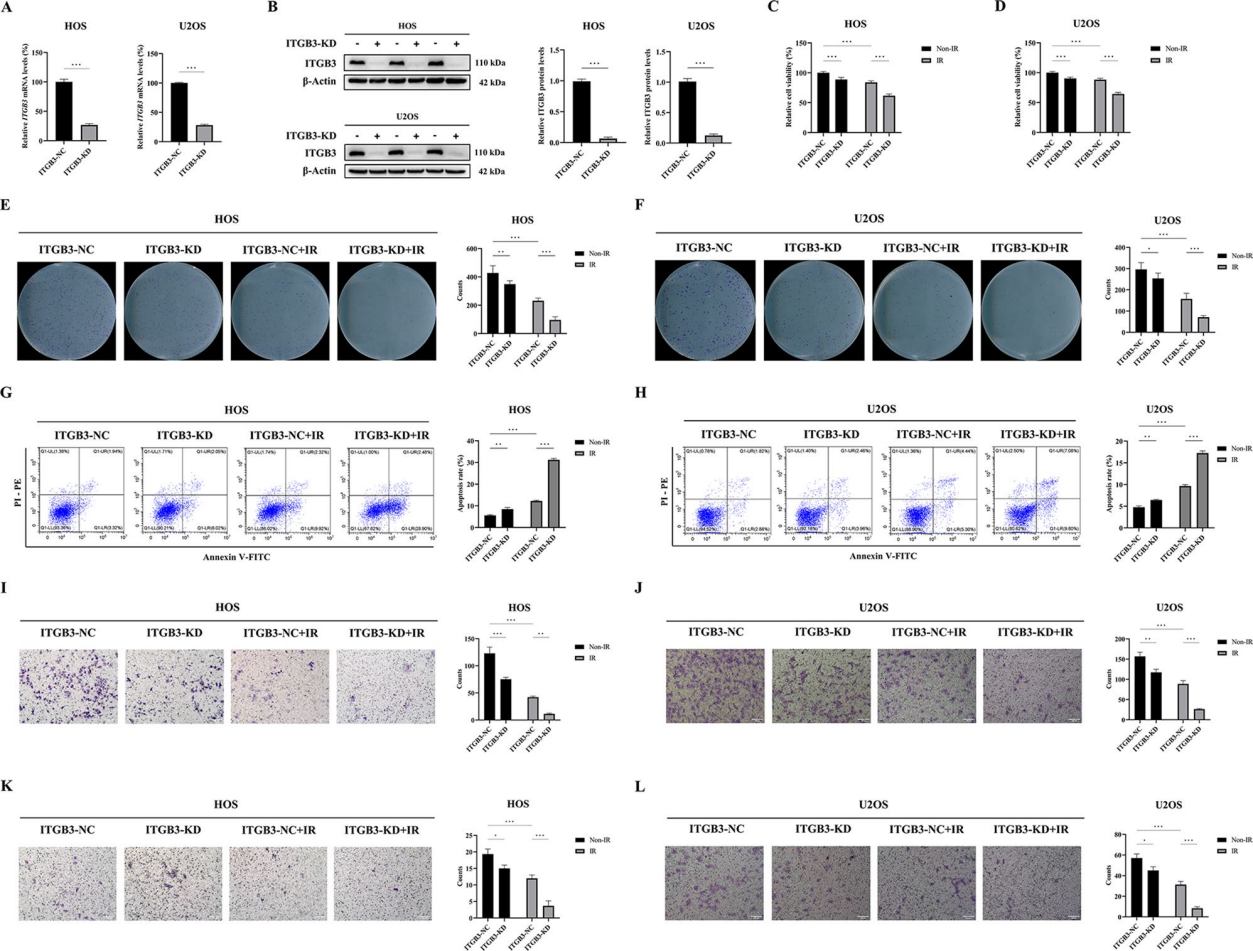
Knockdown of ITGB3 exerts a radiosensitizing effect on osteosarcoma cells in vitro. HOS and U2OS osteosarcoma cells were transfected with lentivirus to generate long-term ITGB3-knockdown (ITGB3-KD) and ITGB3-negative control (ITGB3-NC) lines for subsequent experimentation. The qPCR and western blot were performed to evaluate the mRNA level (**A**) and protein level (**B**) of ITGB3 in HOS and U2OS cells. The cell viability of HOS (**C**) and U2OS (**D**) cells was assessed 24 h posttreatment with 8 Gy of irradiation (IR) or non-IR by Cell Counting Kit-8 assay (*n* = 6 per group). Colony formation assays were conducted by seeding 600 HOS (**E**) and U2OS (**F**) cells per well in 6-well plates, followed by 4 Gy of IR or non-IR treatment. After 7 days, colonies were fixed, stained, and counted (*n* = 6 per group). The apoptosis rates of HOS (**G**) and U2OS (**H**) cells were determined 24 h after 8 Gy of IR or non-IR treatment using flow cytometry (*n* = 3 per group). Migration assays were performed using Transwell inserts. HOS (**I**) and U2OS (**J**) cells were incubated for 12 h before 8 Gy of IR or non-IR treatment. The migrated cells were fixed, stained 12 h posttreatment, and photographed using a microscope at 100x magnification (*n* = 3 per group). Invasion assays were performed using Transwell inserts (coated with Matrigel) in HOS (**K**) and U2OS (**L**) cells, following the same procedure as for the migration assays. *P<0.05, **P<0.01, ***P<0.001, ns = not significant

The results presented above initially suggested the potential of ITGB3-KD to radiosensitize osteosarcoma cells. To systematically validate this radiosensitization effect, we employed lentiviral transfection to establish long-term ITGB3-KD and ITGB3-NC HOS and U2OS osteosarcoma cell models. The successful downregulation of *ITGB3* mRNA expression (Fig. 2A) and ITGB3 protein levels (Fig. 2B) in ITGB3-KD HOS and U2OS cells confirmed the successful establishment of these cellular models.

The results of the CCK-8 assay revealed that, in HOS and U2OS cells, ITGB3-KD alone had a weaker inhibitory effect on cell viability than did IR, whereas the combination of ITGB3-KD and IR considerably suppressed cell viability (Fig. 2C, D). Similarly, in both cell lines, compared with irradiation, ITGB3-KD alone had a less significant effect on reducing the number of colonies formed. However, when combined, the treatments markedly inhibited colony formation, indicating a decreased cell proliferation capacity. (Fig. 2E, F). ITGB3-KD alone induced apoptosis less effectively than did IR, but the combination of ITGB3-KD and IR markedly increased apoptosis (Fig. 2G, H). Additionally, in both HOS and U2OS cells, fewer migrated cells were observed in the ITGB3-KD group than in the ITGB3-NC group, and the combination of ITGB3-KD and IR notably reduced the number of migrated cells (Fig. 2I, J). Comparable trends were observed in invaded cells, indicating similar effects on cell migration and invasion (Fig. 2K, L). Statistical analysis confirmed the synergistic effects between ITGB3-KD and IR in all the aforementioned experiments.

The results of the cell cycle assay revealed that IR induced a “V”-shaped trend in the proportion of osteosarcoma cells in the G1 phase, with an initial decrease followed by an increase within 24 h post-IR. Notably, ITGB3-KD partially counteracted this effect (Fig. S1A). Further statistical analysis revealed that regardless of the time point and combination with IR, ITGB3-KD consistently induced G1-phase arrest, which manifested as an increased proportion of cells in the G1 phase (Fig. S1B).

### Osteogenic differentiation induced by ITGB3 knockdown underlies radiosensitization in osteosarcoma

**Fig. 3:**
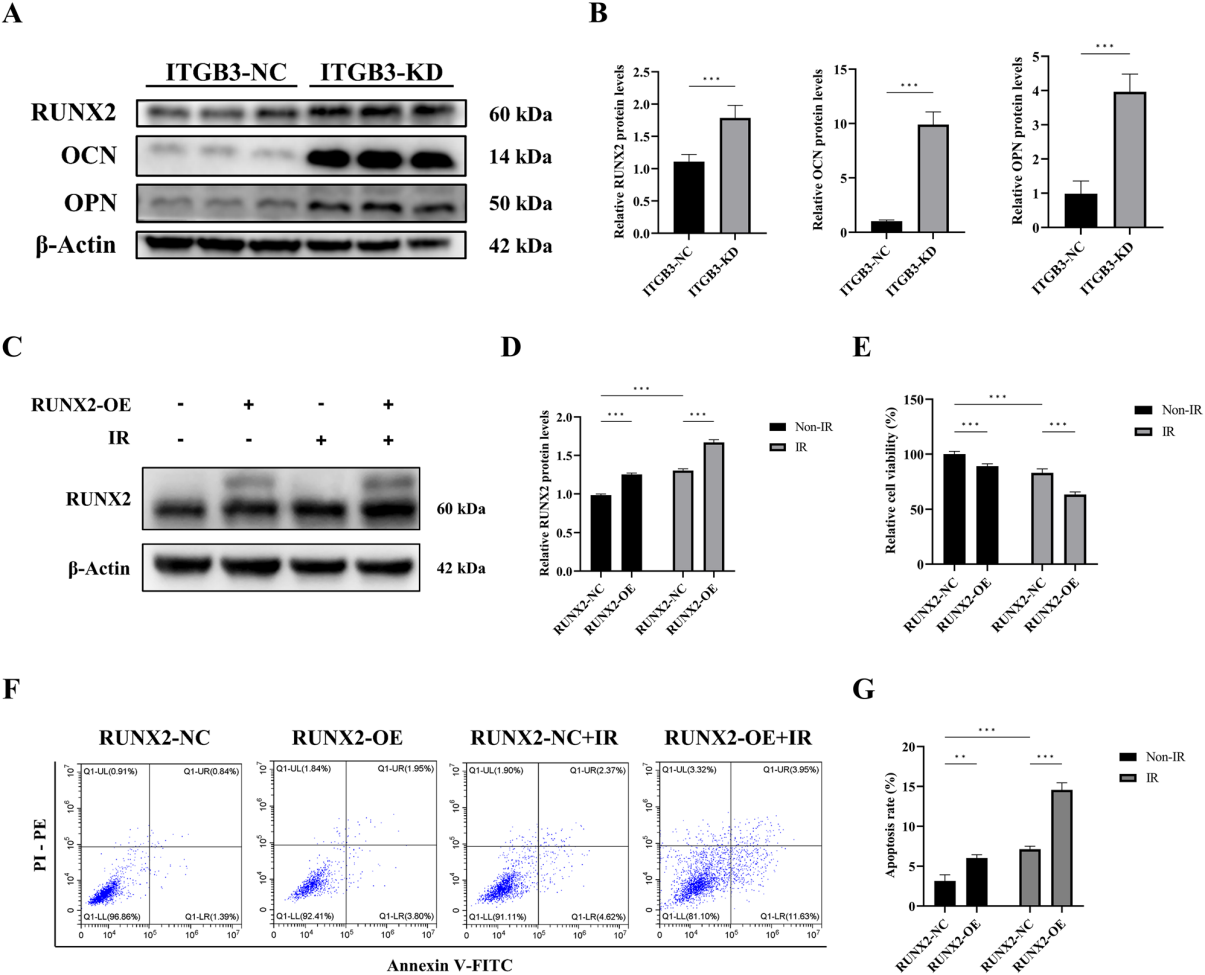
Osteogenic differentiation enhances the radiosensitivity of osteosarcoma cells. The protein levels of the osteogenic markers RUNX2, OCN, and OPN in ITGB3-knockdown (ITGB3-KD) and ITGB3-negative control (ITGB3-NC) HOS cells were evaluated (**A**, **B**). HOS cells were subjected to 8 Gy of irradiation (IR) or non-IR treatment 24 h after RUNX2 overexpression (OE) or negative control plasmid transfection. The RUNX2 protein level (**C**, **D**), cell viability (**E**), and apoptosis rate (**F**, **G**) of cells 24 h posttreatment were detected using western blotting, Cell Counting Kit-8 assay (*n* = 6 per group), and flow cytometry (*n* = 3 per group). *P < 0.05, **P < 0.01, ***P < 0.001, ns = not significant

On the basis of the research background regarding the role of ITGB3 in regulating osteogenic differentiation, we hypothesize that ITGB3 may influence the radiosensitivity of osteosarcoma by modulating osteogenic differentiation. To investigate this, we assessed the impact of ITGB3-KD on the protein levels of the osteogenic markers RUNX2, OCN, and OPN in HOS cells. Our results revealed that ITGB3-KD significantly upregulated the expression of these proteins (Fig. 3A, B). To preliminarily assess the association between osteogenic differentiation status and radiosensitization, we utilized RUNX2 overexpression plasmids to increase the RUNX2 protein level in HOS cells, thereby increasing their osteogenic differentiation capacity (Fig. 3C, D). Following treatment of HOS cells with high RUNX2 expression and negative controls with and without IR, we found that increased osteogenic differentiation (based on RUNX2 overexpression) promoted radiosensitization by inhibiting cell viability (Fig. 3E) and promoting apoptosis (Fig. 3F, G). On the basis of these observations, our subsequent research efforts will focus on elucidating the regulatory role of ITGB3 knockdown in osteogenic differentiation and comprehensively characterizing the role of osteogenic differentiation in the radiosensitization of osteosarcoma.

### ITGB3 knockdown promotes the osteogenic differentiation of osteosarcoma cells by activating the JNK/c-JUN/RUNX2 pathway

Pathways closely associated with osteogenic differentiation include the mitogen-activated protein kinase (MAPK) pathway, the Wnt/β-catenin pathway, the bone morphogenetic protein (BMP) pathway, the Hedgehog pathway, and the NOTCH pathway [27]. To identify potential downstream pathways regulated by ITGB3-KD, we examined the mRNA expression of core molecules within these pathways. Our initial findings revealed that *c-JUN N-terminal kinase 1* (*JNK1)* and *JNK2* mRNAs were upregulated in both HOS and U2OS cells following ITGB3-KD (Fig. S2A). However, *P38*, *extracellular signal-regulated kinase 1* (*ERK1)*, and *ERK2 *mRNAs, which are part of the MAPK pathway, did not display a consistent alteration trend in either cell line (Fig. S2B, C). Similarly, the core molecules of the other osteogenic differentiation pathways, namely, *β-catenin*, *BMP2*, *BMPR2*, *SMO*, *NOTCH1*, and *NOTCH2* mRNAs, also failed to exhibit a consistent alteration trend due to ITGB3-KD in both HOS and U2OS cells (Fig. S2D-G).

Having preliminarily identified JNK as a key downstream molecule that promotes osteogenic differentiation in response to ITGB3-KD, we delved deeper into the alterations in the expression of the JNK protein and its phosphorylated form, along with its well-established downstream target c-JUN protein and its phosphorylated form, under the influence of ITGB3-KD and/or IR. Furthermore, we employed a JNK inhibitor (SP600125, JNKi) in rescue experiments to elucidate the regulatory effects of the JNK/c-JUN pathway on its downstream pathways and effects.

**Fig. 4:**
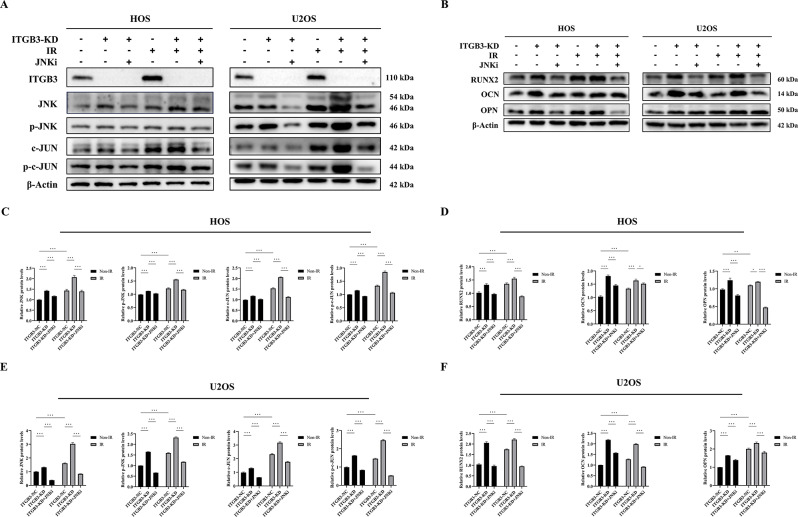
Knockdown of ITGB3 promotes osteogenic differentiation in osteosarcoma by activating the JNK/c-JUN/RUNX2 pathway. ITGB3-knockdown (ITGB3-KD) and ITGB3-negative control (ITGB3-NC) HOS and U2OS cells were subjected to either 8 Gy of irradiation (IR) or non-IR treatment, and additional groups were established via the application of a JNK inhibitor (SP600125, JNKi) 4 h before the IR or non-IR treatment to perform rescue experiments. Protein samples were collected 24 h posttreatment for western blot analysis to explore the regulatory effect of ITGB3-KD on the JNK/c-JUN pathway (**A**, **C**, and **E**) and whether the JNK/c-JUN pathway directly regulated osteogenic differentiation (**B**, **D**, and **F**) in HOS and U2OS cells. *P<0.05, **P<0.01, ***P<0.001, ns = not significant

In both HOS and U2OS cells, IR increased ITGB3 protein levels (Fig. 4A), which aligns with previous observations of *ITGB3* mRNA expression upregulation post-IR in RNA-seq and qPCR experiments (Fig. 1C, D). Furthermore, as shown in Fig. 4A, C, and E, both ITGB3-KD alone and IR alone increased the protein levels of JNK, phosphorylated JNK (p-JNK), c-JUN, and phosphorylated c-JUN (p-c-JUN). Notably, when ITGB3-KD was combined with IR, these proteins exhibited notable upregulation. JNKi application effectively countered this upregulation, confirming its efficacy in subsequent rescue experiments. Additional statistical analysis revealed that, regardless of JNKi application, ITGB3-KD and IR synergistically increased the protein levels of JNK, p-JNK, c-JUN, and p-c-JUN.

After clarifying the synergistic activation of the JNK/c-JUN pathway by ITGB3-KD and IR, we examined the protein levels of the osteogenic markers RUNX2, OCN, and OPN under various intervention scenarios to explore the regulatory role of the JNK/c-JUN pathway in osteogenic differentiation. As shown in Fig. 4B, D, and F, in both HOS and U2OS cells, either ITGB3-KD or IR alone was sufficient to increase the protein levels of RUNX2, OCN, and OPN. Notably, JNKi counteracted the upregulation of RUNX2, OCN, and OPN expression caused by JNK/c-JUN pathway activation. When ITGB3-KD was coupled with IR, RUNX2 protein expression further increased, mirroring the activation trend of the JNK/c-JUN pathway.

These western blot findings suggested that ITGB3-KD enhanced osteogenic differentiation in osteosarcoma by activating the JNK/c-JUN pathway, with RUNX2 serving as the major downstream molecule mediating this effect.

### Knockdown of ITGB3 radiosensitizes osteosarcoma cells by promoting apoptosis through JNK/c-JUN pathway activation

After clarifying the synergistic activation of the JNK/c-JUN pathway by ITGB3-KD and IR, we further examined, under the same intervention and grouping conditions, the changes in cellular apoptosis rates and the levels of the proapoptotic proteins BAX, caspase-3, and cleaved caspase-3 (its active form) in HOS and U2OS cells. This was done to assess the regulatory effect of the JNK/c-JUN pathway activated by ITGB3-KD on the apoptotic pathway. JNKi was utilized to perform rescue experiments.

**Fig. 5:**
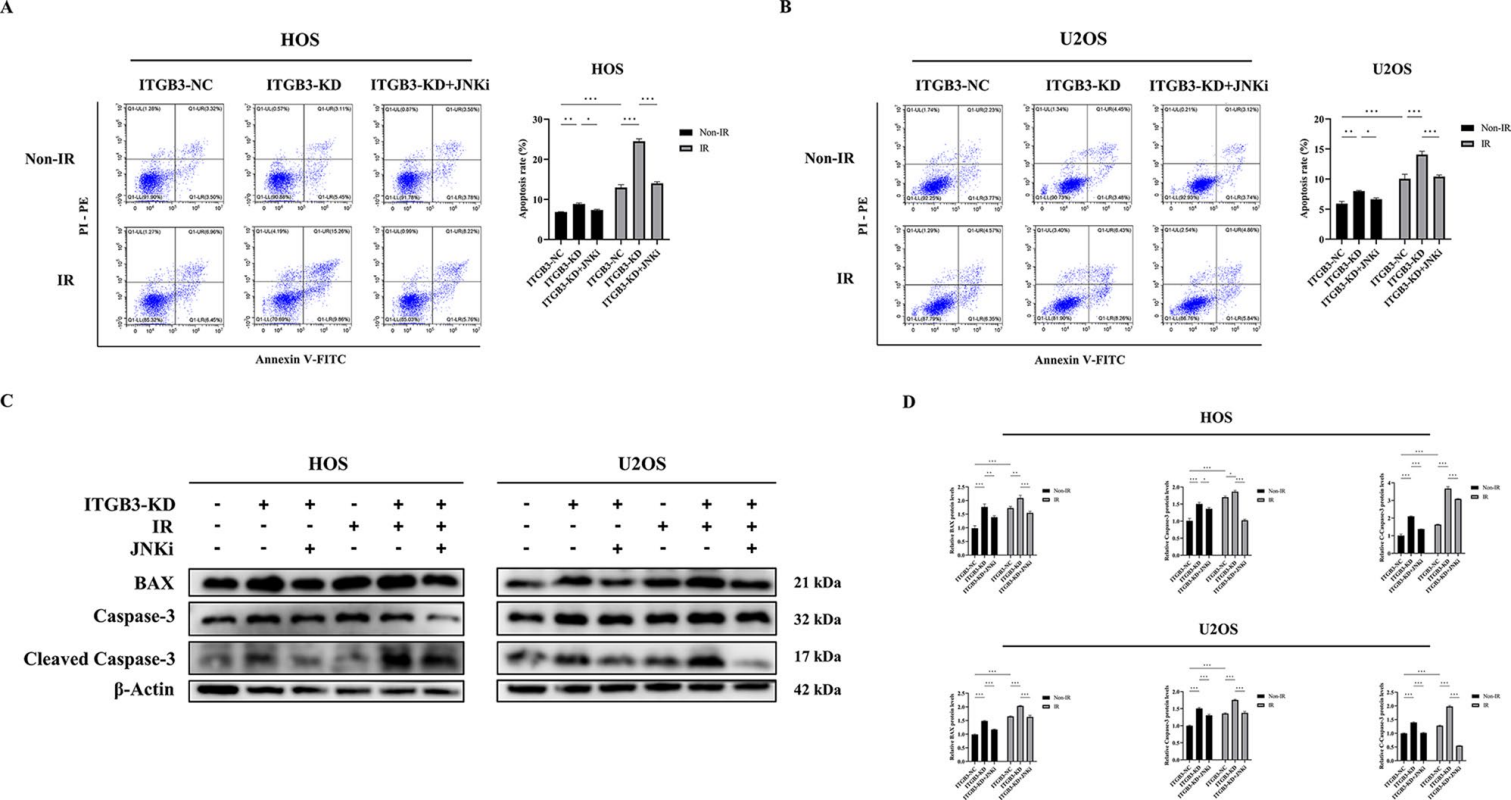
Knockdown of ITGB3 radiosensitizes osteosarcoma cells by promoting apoptosis through activating the JNK/c-JUN pathway. ITGB3-knockdown (ITGB3-KD) and ITGB3-negative control (ITGB3-NC) HOS and U2OS cells were subjected to either 8 Gy of irradiation (IR) or non-IR treatment, and additional groups were established in which a JNK inhibitor (SP600125, JNKi) was applied 4 h before the IR or non-IR treatment to perform rescue experiments. The apoptosis rates in HOS (**A**) and U2OS (**B**) cells were determined 24 h posttreatment using flow cytometry (*n* = 3 per group). Protein samples were collected 24 h posttreatment for western blot analysis to explore the regulatory effect of the JNK/c-JUN pathway on apoptosis (**C**, **D**) in HOS and U2OS cells. *P<0.05, **P<0.01, ***P<0.001, ns = not significant

As shown in Fig. 5A and B, in both HOS and U2OS cells, ITGB3-KD or IR alone increased the level of cellular apoptosis. A remarkable increase in apoptosis was observed when ITGB3-KD was combined with IR. JNKi antagonized the increase in apoptosis promoted by activation of the JNK/c-JUN pathway. As depicted in Fig. 5C and D, ITGB3-KD or IR alone increased the expression levels of the proapoptotic proteins BAX, caspase-3, and cleaved caspase-3. When ITGB3-KD was coupled with IR, the expression levels of these proteins were further elevated. JNKi inhibited the upregulation of these proapoptotic proteins caused by activation of the JNK/c-JUN pathway.

Based on the aforementioned findings, we propose that ITGB3-KD exerts a radiosensitizing effect on osteosarcoma cells by promoting apoptosis via activation of the JNK/c-JUN pathway. However, further validation is needed to determine whether the increased osteogenic differentiation in osteosarcoma cells resulting from ITGB3-KD-mediated activation of the JNK/c-JUN/RUNX2 pathway directly regulates apoptosis in osteosarcoma cells.

### Enhanced osteogenic differentiation status of osteosarcoma cells exerts a radiosensitizing effect by promoting apoptosis

**Fig. 6:**
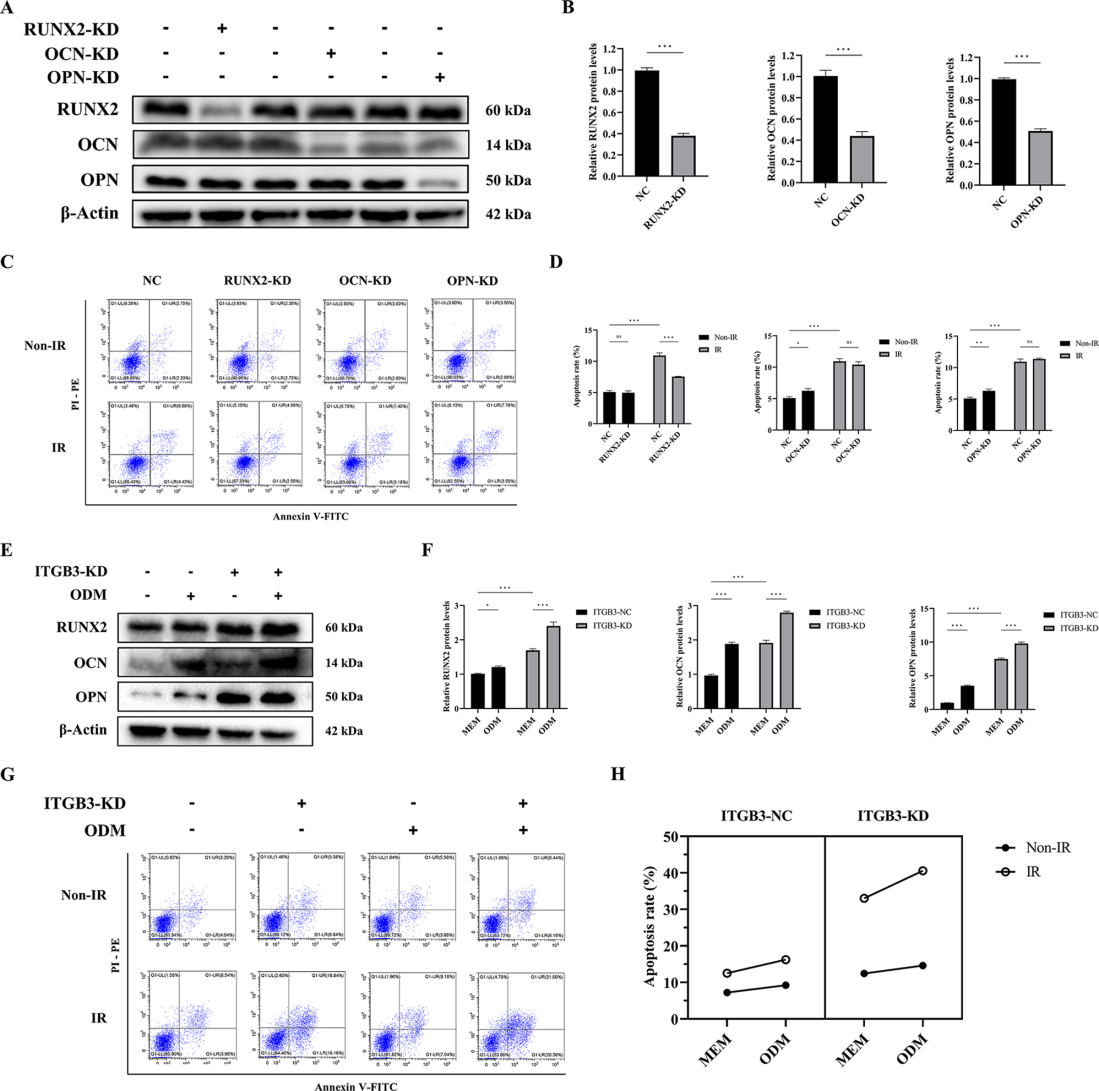
Osteogenic differentiation exerts a radiosensitizing effect on osteosarcoma cells by promoting apoptosis. ITGB3-knockdown (ITGB3-KD) HOS cells were transfected with RUNX2, OCN, OPN, or negative control (NC) siRNA, and western blotting was performed to confirm the knockdown effect (**A**, **B**). The above HOS cells were subjected to either 8 Gy of irradiation (IR) or non-IR treatment, and the apoptosis rate in all groups was determined 24 h posttreatment using flow cytometry, with *n* = 3 per group (**C**, **D**). To further elucidate the radiosensitizing role of osteogenic differentiation, osteogenic differentiation medium (ODM) was utilized to induce a high osteogenic differentiation status in HOS cells, with or without ITGB3-KD. Western blotting was performed to assess the protein levels of osteogenic markers (**E**, **F**). The ITGB3-KD and ITGB3-NC HOS cells, cultured with minimum essential medium (MEM, as control) or ODM for 10 days, were subjected to either 8 Gy of IR or non-IR treatment. The apoptosis rates of the above cells were determined 24 h posttreatment using flow cytometry, with *n* = 3 per group (**G**). Furthermore, the interactions among ITGB3-KD, ODM, and IR were analyzed and demonstrated (**H**). *P<0.05, **P<0.01, ***P<0.001, ns = not significant

We confirmed that ITGB3-KD upregulates the expression of the osteogenic markers RUNX2, OCN, and OPN. Given that these proteins, especially RUNX2, also promote osteogenesis [28–30], we transfected ITGB3-KD HOS cells with RUNX2, OCN, OPN, and negative control (NC) siRNAs to investigate whether inhibiting osteogenic differentiation could counteract the radiosensitizing effect of ITGB3-KD in enhancing apoptosis. As demonstrated in Fig. 6A and B, transfection with RUNX2, OCN, and OPN siRNAs effectively downregulated the expression levels of their respective proteins, indicating that the necessary cellular models were successfully established and suitable for conducting rescue experiments.

As shown in Fig. 6C and D, in the absence of IR, RUNX2-KD did not significantly affect cell apoptosis, whereas OCN-KD and OPN-KD slightly increased apoptosis, potentially because of siRNA-induced toxicity. However, upon exposure to IR, RUNX2-KD counteracted the increase in apoptosis in ITGB3-KD HOS cells, whereas OCN-KD and OPN-KD did not influence apoptosis. These observations indicate that RUNX2 is a downstream molecule through which ITGB3-KD exerts its proapoptotic radiosensitizing effects in osteosarcoma.

To further determine whether the enhancement of osteogenic differentiation mediates a proapoptotic radiosensitizing effect in osteosarcoma, we used osteogenic differentiation medium (ODM) to cultivate ITGB3-KD and ITGB3-NC HOS cells for 10 days to promote osteogenic differentiation. Simultaneously, we established a control group using minimum essential medium (MEM). Both ITGB3-KD and ODM alone elevated the protein levels of RUNX2, OCN, and OPN. When these two factors were applied in combination, they further augmented the expression of these proteins (Fig. 6E, F), indicating the successful construction of a cellular model with a high osteogenic differentiation status on the basis of osteogenic differentiation culture, independent of ITGB3-KD.

Using the cellular model, we assessed the apoptosis rates in various groups (Fig. 6G, H). Three-way factorial ANOVA, which was used to examine the relationships among ITGB3 expression level, IR exposure, and medium type (Fig. 6H), revealed the following: under IR conditions, ODM alone increased apoptosis regardless of the ITGB3 expression level, and a synergistic effect between ITGB3-KD and ODM was evident. Under both ITGB3-NC and ITGB3-KD conditions, ODM alone enhanced apoptosis, and the combination of ODM with IR significantly promoted apoptosis, demonstrating a synergistic effect. These statistical results suggest that ODM-based osteogenic differentiation culture exerted a radiosensitizing effect independent of the ITGB3 expression level, with optimal effects observed when ODM was combined with ITGB3-KD.

Integrating the findings from the aforementioned mechanism investigations with those of a series of rescue experiments and osteogenic differentiation cultures, we have substantiated that ITGB3-KD potentiates osteogenic differentiation and exerts proapoptotic radiosensitizing effects in osteosarcoma by activating the JNK/c-JUN/RUNX2 pathway.

### Knockdown of ITGB3 enhances the radiosensitivity of osteosarcoma cells in vivo by activating the JNK/c-JUN/RUNX2 pathway

Using HOS human osteosarcoma cells transfected with ITGB3-KD and ITGB3-NC lentiviruses, we constructed BALB/c nude mouse models of subcutaneous tumors in the inguinal region and orthotopic tumors in the tibia for in vivo efficacy and mechanistic validation. In the subcutaneous tumor model, groups treated with JNKi were established for rescue experiments.

**Fig. 7:**
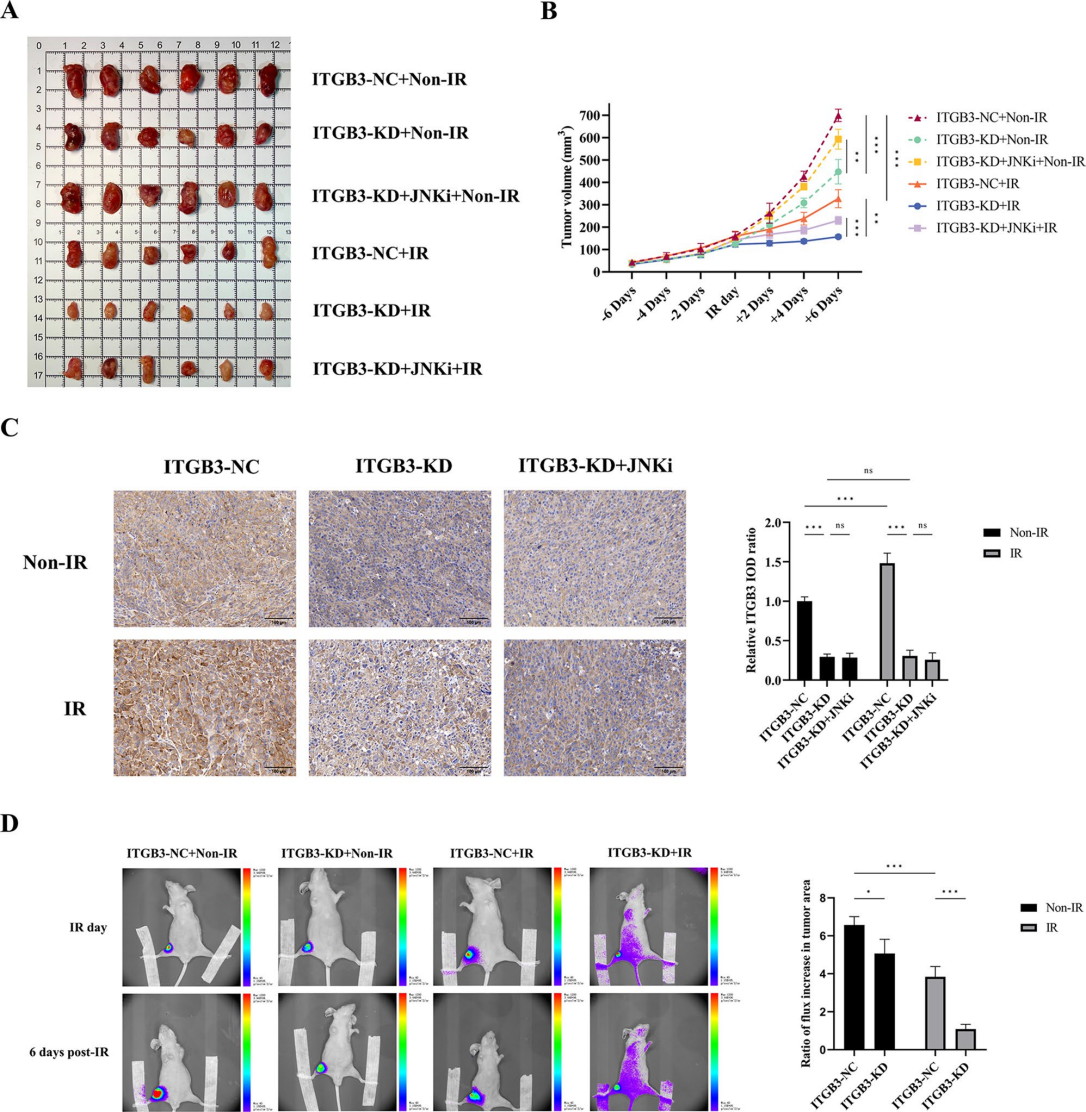
Knockdown of ITGB3 exerts a radiosensitizing effect in osteosarcoma mouse models. ITGB3-knockdown (ITGB3-KD) and ITGB3-negative control (ITGB3-NC) HOS cells were injected into the inguinal subcutaneous region of BALB/c nude mice to establish a subcutaneous tumor model. Additionally, groups of mice receiving intraperitoneal injections of the JNK inhibitor (SP600125, JNKi) were established, with injections administered 24 h before, 4 h before, and daily after 20 Gy of local irradiation (IR) or non-IR treatment. Six days after receiving IR or non-IR treatment, mice were euthanized. Subsequently, the subcutaneous tumors were photographed (**A**), and the growth curves of the tumors were plotted, with *n* = 6 per group (**B**). ITGB3 immunohistochemical staining was performed on subcutaneous tumor specimens from each group, and images were captured at 200x magnification (**C**). Statistical analysis of ITGB3 expression levels was conducted using the integrated optical density (IOD) method, with *n* = 3 per group. Furthermore, ITGB3-KD and ITGB3-NC HOS cells were injected into the tibial bone marrow cavity to establish an orthotopic tibial tumor model in BALB/c nude mice. The first bioluminescence measurement was conducted before 20 Gy of local IR or non-IR treatment. On the 6th day after treatment, bioluminescence measurement was repeated to calculate the ratio of bioluminescent flux increase in the tumor area of each mouse, reflecting tumor growth. Typical images are presented (**D**). Note: The bioluminescent flux scale has been standardized. *P<0.05, **P<0.01, ***P<0.001, ns = not significant

In the subcutaneous tumor model, ITGB3-KD alone mildly inhibited tumor growth, whereas the combination of ITGB3-KD with IR notably suppressed tumor growth. The JNKi alleviated the tumor growth inhibition effect induced by ITGB3-KD alone or ITGB3-KD combined with IR (Fig. 7A, B). ITGB3 expression in the ITGB3-NC + IR group was greater than that in the ITGB3-NC + non-IR group, and all ITGB3-KD groups exhibited low ITGB3 expression, suggesting stable differential expression of ITGB3 in the subcutaneous tumor model (Fig. 7C). This ITGB3 expression pattern was consistent with the results of the western blot analysis conducted in vitro (Fig. 4A). In the orthotopic tibial tumor model, we further validated the radiosensitizing effect of ITGB3-KD in vivo using bioluminescence measurements. Similar to the subcutaneous tumor model, ITGB3-KD alone mildly inhibited tumor growth, whereas the combination of ITGB3-KD with IR resulted in significant suppression of tumor growth (Fig. 7D).

**Fig. 8:**
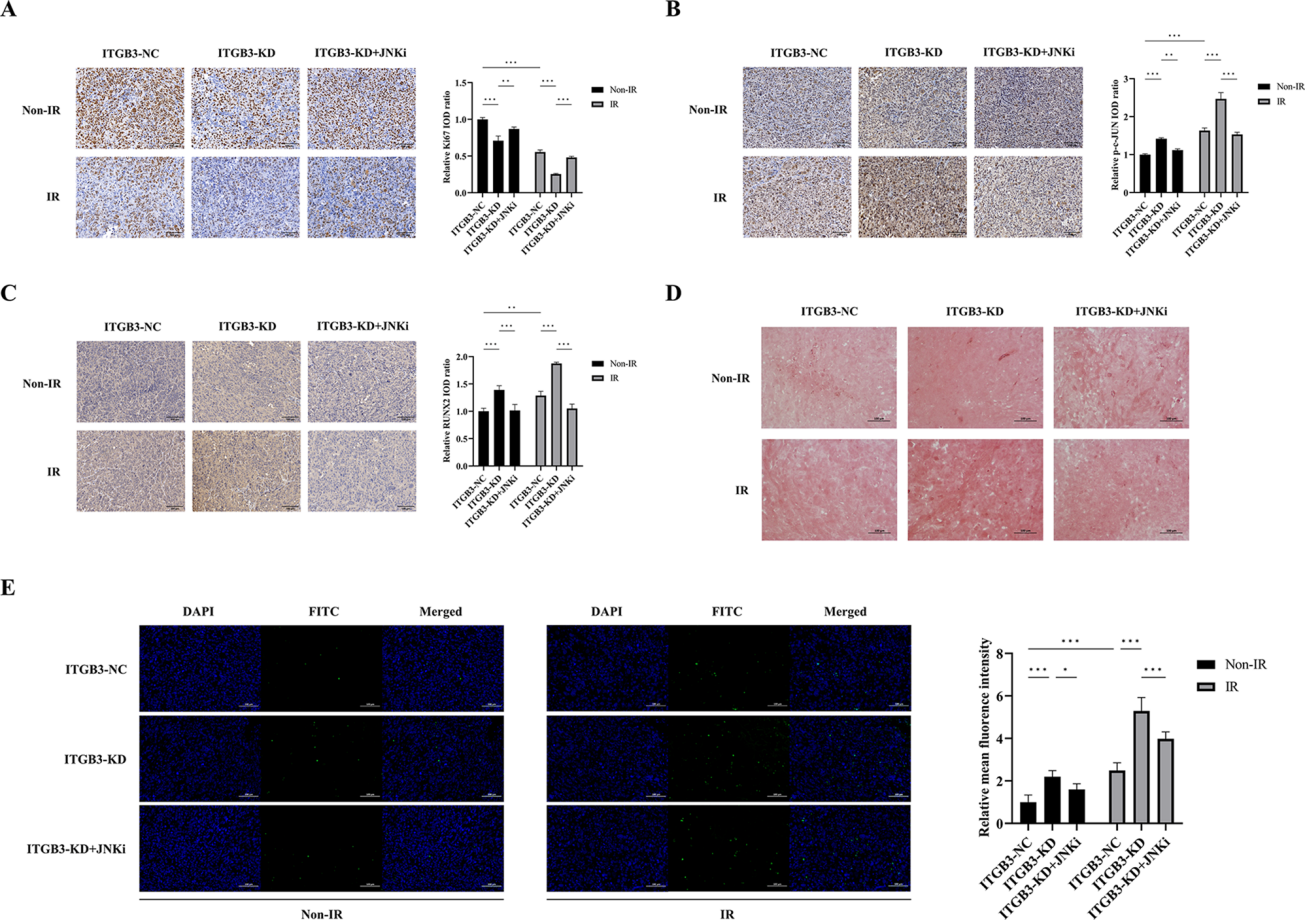
Knockdown of ITGB3 exerts radiosensitizing effects in vivo by activating the JNK/c-JUN/RUNX2 pathway. Immunohistochemical staining for Ki67 (**A**), p-c-JUN (**B**), and RUNX2 (**C**) was performed on subcutaneous tumor specimens from each group, and images were captured at 200x magnification. Statistical analysis of Ki67, p-c-JUN, and RUNX2 expression levels was conducted using the integrated optical density (IOD) method, with *n* = 3 per group. Alizarin red S staining was performed on subcutaneous tumor specimens from each group, with *n* = 3 per group, and specimens were imaged at 200x magnification (**D**). TUNEL staining was carried out on the subcutaneous tumor specimens from each group; FITC was used to stain apoptotic cells, and DAPI was used to stain cell nuclei. Images were individually captured using the FITC and DAPI channels of a fluorescence microscope at 200x magnification. In addition to the merged images, individual FITC and DAPI channel images were presented, and a quantitative analysis was conducted, with *n* = 3 per group (**E**). *P<0.05, **P<0.01, ***P<0.001, ns = not significant

Subcutaneous tumor specimens were subjected to immunohistochemical (IHC) staining for Ki67, p-c-JUN, and RUNX2 proteins, along with TdT-mediated dUTP nick-end labeling (TUNEL) staining for apoptosis detection. Both ITGB3-KD and IR resulted in decreased Ki67 expression, with a more pronounced reduction when these two treatments were combined, suggesting the suppression of proliferative capacity (Fig. 8A). The application of JNKi reversed the ITGB3-KD-induced downregulation of Ki67 expression. IHC staining for p-c-JUN served as an indicator of JNK/c-JUN pathway activation. Both ITGB3-KD and IR upregulated p-c-JUN expression, and the combination of these treatments led to notable upregulation (Fig. 8B). The JNKi counteracted the ITGB3-KD-induced upregulation of p-c-JUN. Similarly, both ITGB3-KD and IR upregulated RUNX2 expression, with a marked increase observed when combined (Fig. 8C). The JNKi also mitigated the ITGB3-KD-induced upregulation of RUNX2. The TUNEL assay revealed that both ITGB3-KD and IR promoted apoptosis and that their combined effect was additive, significantly enhancing apoptosis (Fig. 8E). The JNKi mitigated the ITGB3-KD-induced apoptotic effect. These findings suggest that ITGB3-KD activated the JNK/c-JUN/RUNX2 pathway and promoted apoptosis in vivo, which aligns with the results of the in vitro experiments. To assess whether the ITGB3-KD-induced increase in osteogenic differentiation of osteosarcoma cells led to extensive mineralization at the late stage of osteogenic differentiation, alizarin red S (ARS) staining was conducted. Compared with that in the ITGB3-NC + non-IR group, the increase in brown‒red mineralized nodules in the ITGB3-KD + non-IR group was limited, and there was no substantial increase in mineralized nodules in the ITGB3-KD + IR group (Fig. 8D).

## Discussion

Currently, research on radiosensitization in osteosarcoma is still limited, with no widely accepted molecular targets identified; consequently, no radiosensitizing drugs for osteosarcoma have been commercialized to date. Through integrative analysis involving RNA-seq screening, literature curation, expression validation, and functional characterization, we identified ITGB3 as a putative radiosensitization target in osteosarcoma. Notably, while several studies have reported the radiosensitizing effect of the broad-spectrum αvβ3 and αvβ5 inhibitor cliengitide trifluoroacetate in malignancies [25, 26], cilengitide trifluoroacetate simultaneously inhibits both integrin αvβ3 and αvβ5 while leaving integrin α2bβ3 unaffected. This complicates the determination of the individual contributions of ITGB3, ITGAV, and ITGB5. With respect to ITGB3 specifically, despite previous studies showing that it confers resistance to cisplatin in osteosarcoma [24] and demonstrating the tumor-suppressing impact of ITGB3-KD [31–33], the role of ITGB3 in the radiosensitization of malignancies remains largely unexplored.

We comprehensively validated the radiosensitizing effect of ITGB3-KD on osteosarcoma cells in vitro by performing assays for cell viability, proliferation, apoptosis, migration, and invasion. Furthermore, both subcutaneous and orthotopic tibial tumor models revealed the radiosensitizing effect of ITGB3-KD on osteosarcoma in vivo. Additionally, we confirmed that ITGB3-KD slightly inhibits G1 phase progression. Although cell cycle arrest enhances the radiosensitization of malignancies through the promotion of apoptosis [34, 35], owing to the relatively weak inhibitory effect of ITGB3-KD on the G1 phase, we hypothesize that a more pivotal mechanism is involved in facilitating the radiosensitization of osteosarcoma cells, potentially by promoting apoptosis and other biological processes.

Given that osteosarcoma has osteogenic differentiation potential, cells at different stages of differentiation exhibit variable sensitivity to IR, and ITGB3 has the potential to modulate osteogenic differentiation, we delved into the mechanism underlying the radiosensitizing effect of ITGB3-KD in osteosarcoma, particularly its influence on osteogenic differentiation. Quantitative assessment of osteogenic markers (RUNX2, OCN, and OPN) confirmed that ITGB3-KD drives osteogenic differentiation in osteosarcoma. To functionally validate this phenotype, we engineered RUNX2-overexpressing osteosarcoma cells that exhibited increased osteogenic differentiation. Radiosensitivity assays combining cell viability analysis with apoptosis quantification demonstrated that differentiated cells presented a significantly increased response to radiation, establishing a functional interplay between osteogenic maturation and radiosensitization. Prior studies have highlighted CSC-targeted strategies to overcome radioresistance [10–13, 36]. The limited incidence of CSCs in osteosarcoma [37] suggests broader therapeutic potential in the induction of differentiation. Particularly in cases of poorly differentiated osteosarcoma, increasing the global differentiation status may represent a more impactful radiosensitization approach than CSC eradication alone.

The JNK/c-JUN pathway is a multifaceted signaling cascade that regulates cellular apoptosis and autophagy and plays a crucial role in stem cell differentiation and embryonic development [38–40]. Relevant studies have suggested that activation of the JNK/c-JUN pathway in response to IR stimulation promotes tumor cell apoptosis [41, 42]. Furthermore, studies have revealed the positive impact of JNK pathway activation on facilitating osteogenic differentiation [43, 44]. To our knowledge, this study provides the first experimental evidence of the synergistic activation of the JNK/c-JUN pathway by ITGB3-KD in combination with IR. Although prior investigations have not explicitly linked ITGB3-KD to this pathway, a study has shown that inhibiting integrin αvβ3 expression upregulates this pathway, thereby supporting our observations [45].

**Fig. 9:**
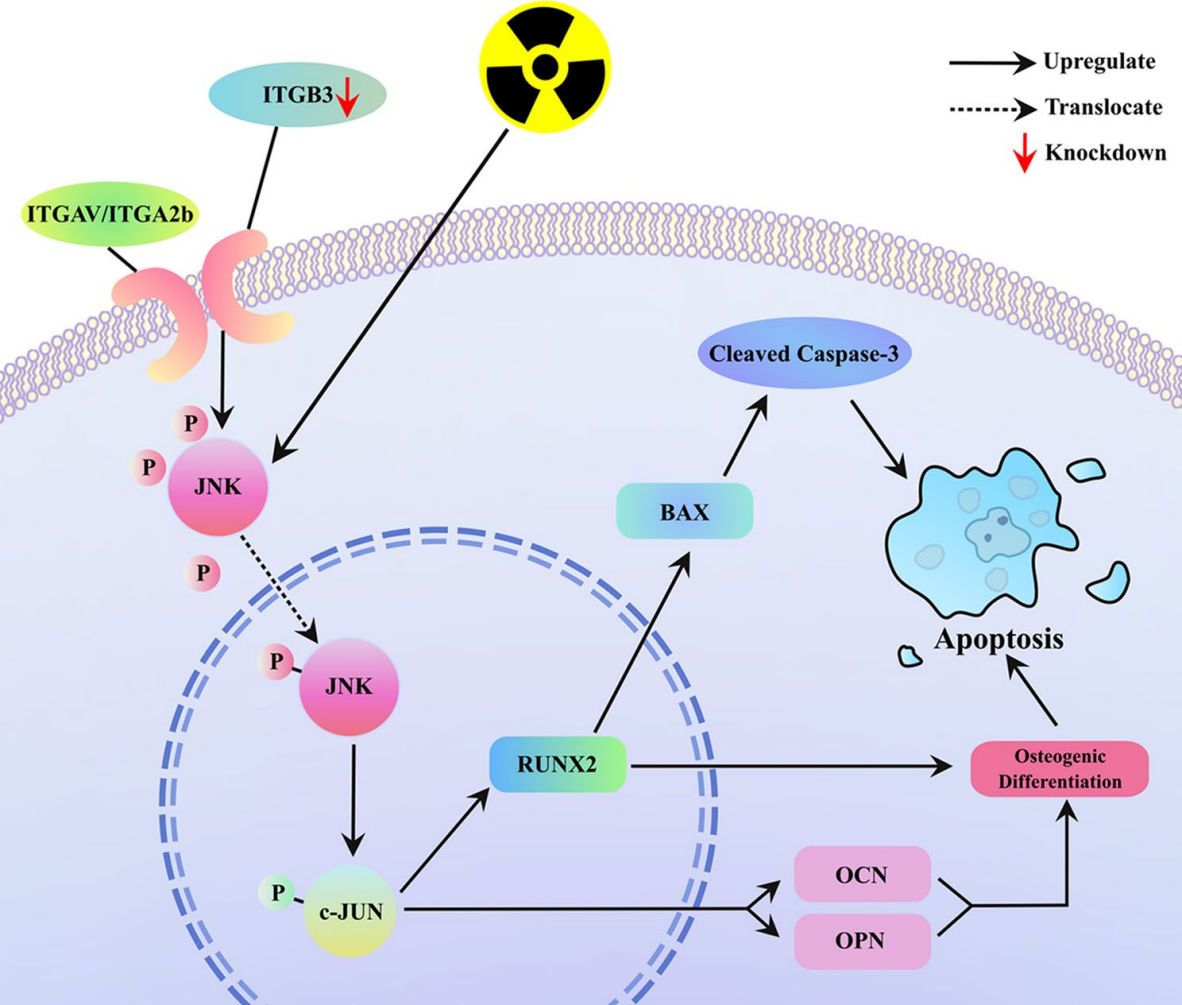
Mechanistic illustration: ITGB3-knockdown enhances osteogenic differentiation and radiosensitizes osteosarcoma cells via JNK/c-JUN/RUNX2 pathway activation. There is a synergistic interaction between ITGB3 knockdown and irradiation in activating the JNK/c-JUN signaling pathway. Upon the elevation of phosphorylated JNK expression, an increase in the expression of phosphorylated c-JUN occurs, which then upregulates osteogenic markers, including RUNX2, OCN, and OPN, thereby suggesting the enhancement of osteogenic differentiation in osteosarcoma cells. Furthermore, the upregulation of RUNX2 promotes apoptosis by activating BAX, which subsequently enhances the expression of cleaved caspase-3, resulting in apoptosis. The osteogenic differentiation enhanced through osteogenic differentiation medium culture also exerts a proapoptotic radiosensitizing effect on osteosarcoma cells

Classical osteogenic differentiation encompasses three major stages: the induction of pluripotent stem cell differentiation by osteogenic factors, the secretion of bone matrix proteins, and calcium deposition and mineralization [46, 47]. RUNX2, a pivotal osteogenic transcription factor, not only functions as an indicator of osteogenic differentiation but also actively promotes it [28]. Through rescue experiments employing JNK inhibitors, we validated the effect of JNK/c-JUN pathway activation on upregulating RUNX2, OCN, and OPN expression, as well as its ability to facilitate apoptosis. Additional rescue experiments using siRNAs further suggested that the upregulation of RUNX2 mediated the apoptotic response. Taken together, these findings indicate that ITGB3-KD exerts radiosensitizing effects on osteosarcoma cells by promoting osteogenic differentiation and apoptosis through activation of the JNK/c-JUN/RUNX2 pathway (Fig. 9). The aforementioned pathway was also validated in a subcutaneous osteosarcoma mouse model. However, ARS staining revealed that the osteogenic differentiation promoted by ITGB3-KD had not yet reached the late stage of extensive calcium deposition and mineralization. This may be attributed to the short culture duration (16 days from implantation to collection), inconsistency of the local microenvironment with the origin site, or deficiency of certain bone matrix proteins.

We further used osteogenic differentiation medium, which is commonly used to induce osteogenic differentiation in bone marrow mesenchymal stem cells, for an extended duration to facilitate osteogenic differentiation in osteosarcoma cells. Intriguingly, our findings revealed that even in the absence of ITGB3-KD, augmenting osteogenic differentiation in osteosarcoma cells could elicit a radiosensitizing effect. Furthermore, when ITGB3-KD was synergistically combined with conditions that promote osteogenic differentiation, the radiosensitizing effect on osteosarcoma cells was notably potentiated. This result further corroborates the beneficial impact of promoting osteogenic differentiation to increase the radiosensitivity of osteosarcoma for therapeutic purposes.

Targeted molecular therapies represent a promising frontier for improving the prognosis of cancer patients [48–50]. While our findings establish targeting ITGB3 as a radiosensitizing strategy in preclinical osteosarcoma models, three key limitations merit discussion. First, the reliance on viral delivery systems poses translational challenges due to immunogenicity risks, though emerging alternatives like cyclic RGD peptide (cRGD)-functionalized exosomes or synthetic nanovesicles [51–53] may offer tumor-targeted siRNA delivery with minimized off-target effects. Second, while this study provides initial evidence that ITGB3-KD promotes osteogenic differentiation and induces radiosensitization in osteosarcoma through activation of the JNK/c-JUN/RUNX2 pathway, the mechanistic crosstalk between osteogenic differentiation and other radiosensitization-related pathways (e.g., DNA damage repair pathways) remains uncharacterized. RNA-seq could be applied to delineate the interplay between osteogenic differentiation and radiosensitization mechanisms. Finally, the exclusive use of immunocompromised models precludes assessment of tumor-immune microenvironment interactions—a critical gap given radiotherapy’s immunomodulatory potential, necessitating future evaluation in immunocompetent orthotopic models to elucidate combinatorial immune-radiation synergies.

## Conclusions

This study demonstrates the radiosensitizing effect of ITGB3-KD on osteosarcoma both in vitro and in vivo. The findings suggest that ITGB3-KD enhances the radiosensitivity of osteosarcoma cells by promoting osteogenic differentiation and apoptosis via activation of the JNK/c-JUN/RUNX2 signaling pathway. Importantly, this study not only implicates ITGB3 as a candidate target for osteosarcoma radiosensitization but also highlights the positive role of osteogenic differentiation in inducing radiosensitivity. These observations warrant further investigation into differentiation-focused combinatorial strategies for osteosarcoma radiotherapy.

## Materials and methods

### Cell culture and cell treatments

The human osteosarcoma cell lines HOS and U2OS (Fuheng Biotech, China) were cultured in minimum essential medium (MEM) and high-glucose Dulbecco’s modified Eagle’s medium (DMEM), respectively, supplemented with 10% fetal bovine serum (FBS, Vivacell, C04001) and 1% penicillin-streptomycin-amphotericin B (Biosharp, BL142).

To examine the role of ITGB3, ITGB3-knockdown (ITGB3-KD) and ITGB3-negative control (ITGB3-NC) lentiviruses were transfected into both cell lines. Transfected cells were then selected with 2 µg/ml puromycin (Solarbio, P8230) and maintained under 1 µg/ml selection pressure.

For osteogenic differentiation culture in HOS cells, osteogenic differentiation medium (Oricell, HUXMX-90021) was used.

In the cell experiments, cells treated with the JNK inhibitor (JNKi) received SP600125 (Selleck, S1460) at a final concentration of 10 µM, which was introduced into the culture medium 4 h before the application of either irradiation (IR) or non-IR treatments.

### Mice and treatments

Five-week-old male BALB/c nude mice were obtained from Jihui Biotechnology, China. For the subcutaneous osteosarcoma model, after one week of acclimatization, 45 mice were randomly divided into the ITGB3-NC (15 mice) and ITGB3-KD (30 mice) groups. Mice in each group received subcutaneous injections in the groin area of 4 million corresponding HOS cells. Once the ITGB3-NC tumors reached an average volume of 100 mm³, the mice were regrouped into six subgroups based on tumor volume and origin, as follows: ① ITGB3-NC + non-IR; ② ITGB3-NC + IR; ③ ITGB3-KD + non-IR; ④ ITGB3-KD + JNKi + non-IR; ⑤ ITGB3-KD + IR; and ⑥ ITGB3-KD + JNKi + IR. Mice with underdeveloped tumors were excluded before treatment. Mice in groups ②, ⑤, and ⑥ received 20 Gy of localized IR. The tumor volumes were continuously monitored. The mice were euthanized when significant differences in subcutaneous tumor volume emerged between the subgroups. Tumors were photographed and harvested for further assays. The subcutaneous tumor volume (mm^3^) was calculated as L * W * W * 0.52, where L is the length of the subcutaneous tumor, and W is the width of the subcutaneous tumor.

SP600125 was administered intraperitoneally to mice in the JNKi-treated groups at 15 mg/kg 4 and 24 h before the IR or non-IR treatments, followed by daily injections to maintain the concentration. The dosing regimen was based on a study of 28-day continuous SP600125 injections in mice [54]. The SP600125 solution was prepared using a 5% dimethyl sulfoxide (DMSO) stock solution, 40% PEG300, 5% Tween 80, and 50% ddH2O, following the manufacturer’s instructions.

After one week of acclimatization, 20 mice were divided into ITGB3-NC (10 mice) and ITGB3-KD (10 mice) groups for the orthotopic tibial osteosarcoma model. The mice received 10-µl injections of 200,000 HOS cells resuspended in Matrigel (Corning, 354277) into the tibial bone marrow cavity. The injection was performed by inserting a 26-G needle from the medial side of the anterior cruciate ligament under knee hyperflexion, puncturing the tibial marrow cavity by rotating the needle, and then withdrawing it. A 10 µl microsyringe with a 30-G needle was inserted through the puncture path to slowly inject 10 µl of cell suspension. The puncture site was gently pressed to prevent leakage and bleeding. The modeling method was adjusted based on existing literature [55, 56]. Once most of the mice exhibited palpable orthotopic tumors, the first bioluminescence measurement was conducted. Mice confirmed to have tumor formation through bioluminescence measurement were randomized into four subgroups on the basis of body weight and cell origin, as follows: ① ITGB3-NC + non-IR (*n* = 3); ② ITGB3-NC + IR (*n* = 4); ③ ITGB3-KD + non-IR (*n* = 3); and ④ ITGB3-KD + IR (*n* = 4). Subsequently, localized treatment with 20 Gy of IR was administered to the tumor sites in groups ② and ④. Six days post-IR, a second bioluminescence measurement was performed.

In this study, isoflurane gas inhalation was used for anesthesia, and at the end of the observation, mice were euthanized via cervical dislocation under isoflurane anesthesia.

### Irradiation (IR)

Cells and mice were irradiated with γ-rays using a ^60^Co radiation source (Naval Medical University, China). The radiation doses were delivered at a rate of 1 Gy per minute, with precise calibration achieved by adjusting the exposure duration. During localized IR in mice, strategic placement of lead shielding between the mouse and the radiation source was employed to protect against unintended exposure to other organs. The irradiation doses employed in both in vivo and in vitro experiments were determined based on existing literature [57, 58] and have been validated in previous studies conducted by our research group [35].

In experiments related to the cell cycle, a series of time points post-IR were used to observe the temporal progression of IR-induced biological effects. The 0-h post-IR time point corresponds to the moment immediately before the IR procedure, ensuring a consistent baseline for subsequent comparisons.

### RNA sequencing (RNA-seq)

HOS cells were subjected to either 8 Gy of IR or non-IR treatment (*n* = 3 for each group), and 12 h later, RNA was extracted using RNAiso Plus (Takara, 9109). Samples were further processed and analyzed by Gene Denova, China. Using the visualization platform Omicsmart, heatmaps for sample correlation analysis, volcano plots for differential gene expression, and heatmaps for differential gene expression were constructed.

### Real-time quantitative PCR (qPCR)

Total RNA was extracted via a Total RNA Extraction Kit (Solarbio, R1200). For cDNA preparation, PrimeScript RT Master Mix (Takara, RR036A) and a reverse transcription machine (Analytik-Jena) were utilized. TB Green Premix Ex Taq II (Takara, RR820A) was subsequently used to prepare the qPCR mixture, which was processed using a QuantStudio 1 PCR System (Thermo Fisher) according to the manufacturer’s instructions. The sequences of primers used in this study are listed in Supplementary Table 1.

### Transfection of siRNA

The siRNAs targeting ITGB3, RUNX2, OPN, and OCN were obtained from Tsingke Biotechnology, China. These siRNAs were utilized at a working concentration of 100 nM, and Lipofectamine 3000 (Thermo Fisher, L3000015) was used as the transfection reagent. The sequences of the siRNAs used in this study are detailed in Supplementary Table 2.

### Lentiviral construction

The lentiviral vector utilized in this study was provided by GeneChem and was designated GV344. The element order of this vector was as follows: hU6-MCS-Ubiquitin-firefly_Luciferase-IRES-puromycin. The negative control vector was designated CON206. This lentiviral system was employed for either knocking down the human ITGB3 protein or conducting negative control experiments. The target sequences used for lentiviral construction are listed in Supplementary Table 3.

### Western blot

Western blot detection was conducted based on the existing literature [59]. Total protein was extracted using RIPA buffer (Beyotime, P0013B). The proteins were fractionated by 10%, 12.5%, or 15% sodium dodecyl sulfate-polyacrylamide gel electrophoresis (SDS‒PAGE; Epizyme) and electrotransferred onto 0.2 µm or 0.45 µm polyvinylidene difluoride (PVDF) membranes (Millipore). Following a 1-hour blocking step with 5% nonfat milk at room temperature, the PVDF membranes were incubated overnight at 4 °C with the primary antibody. Afterward, the membranes were probed with a secondary antibody for 1 h at room temperature. Finally, the immunoblots were developed using an HRP substrate luminol reagent (Immobilon, WBKLS0500) and visualized through enhanced chemiluminescence (ECL) detection systems, such as Gelview 6000plus (Biolight Biotechnology) and ChemiDoc XRS+ (Bio-Rad). The antibodies used for western blotting are listed in Supplementary Table 4.

### Cell viability detection

The viability of cells subjected to various treatments was assessed via a Cell Counting Kit-8 (CCK-8; Dojindo, CK04). Specifically, 24 h posttreatment, the optical density (OD) values were measured at 450 nm with a spectrophotometer (BioTek), with *n* = 6 per group.

### Apoptosis assay

Cells subjected to various treatments were prepared and stained with the Annexin V-FITC/PI Apoptosis Detection Kit (TransGen, FA101-01) 24 h posttreatment (*n* = 3 per group). The stained cells were collected and analyzed by flow cytometry (Beckman) and CytExpert software (Beckman).

### Colony formation assay

The colony formation assay was finely tuned based on existing literature [60]. Six hundred cells were seeded in 6-well plates one day before treatment (*n* = 6 per group). The cells were subjected to either 4 Gy of IR or non-IR treatment. Seven days after the indicated treatment, the cells were fixed with 4% paraformaldehyde and subsequently stained with crystal violet. The number of clones was calculated using ImageJ software (version 1.54d; National Institutes of Health, USA).

### Migration assay

Forty thousand cells (suspended in 100 µl of serum-free medium) were seeded into the upper chambers of Transwell inserts (Corning, 3422), while 600 µl of complete medium was added to the corresponding lower chambers (*n* = 3 per group). The cells were exposed to either 8 Gy of IR or non-IR treatment 12 h after seeding. Twelve hours posttreatment, the cells were fixed with 4% paraformaldehyde and subsequently stained with crystal violet. The inserts were photographed using a microscope (Carl Zeiss) at 100x magnification, and the number of migrated cells was calculated using ImageJ software.

### Invasion assay

The procedure used for the invasion assay was essentially the same as that used for the migration assay, but the Transwell inserts were coated with Matrigel (Corning, 354277).

### Cell cycle assay

HOS cells that had been transfected with either ITGB3-NC or ITGB3-KD lentivirus were subjected to either 8 Gy of IR or non-IR treatment. The cells were subsequently prepared and stained using a Cell Cycle and Apoptosis Analysis Kit (Beyotime, C1052) within 24 h after treatment (*n* = 3 per group). The stained cells were collected and analyzed using flow cytometry (Beckman) and CytExpert software (Beckman).

### Immunohistochemistry (IHC)

Paraffin sections (*n* = 3 per group) were deparaffinized, hydrated, and subjected to antigen retrieval. Endogenous peroxidase was blocked with 3% hydrogen peroxide. The tissues were blocked with 3% bovine serum albumin (BSA, Servicebio, GC305010) for 30 min and incubated with primary antibody at 4 °C overnight, followed by incubation with secondary antibody at room temperature for 1 h. DAB (Servicebio, G1212) staining was performed, and color development was terminated under microscopic observation. Nuclei were counterstained with hematoxylin. After dehydration, the tissues were sealed. Images were captured at 200x magnification, and the integrated optical density (IOD) was quantified using ImageJ software. The antibodies used are listed in Supplementary Table 4.

### Bioluminescence measurement

A 15 mg/mL solution of fluorescein potassium (Yeasen, 40902ES03) was freshly prepared in calcium- and magnesium-free Dulbecco’s phosphate-buffered saline (DPBS) to prevent fluorescence quenching. Mice were subsequently administered 150 mg/kg of the substrate via intraperitoneal injection, followed by bioluminescence imaging 15 min later. Before imaging, the mice were anesthetized with isoflurane to ensure their immobility during the 10-second exposure period. They were then securely positioned on a board with a black background to expose the tibial tumor region. Bioluminescence imaging was conducted using a Bio-Real^®^ In Vivo Bioluminescence Imaging System-3000 (LABATECH GmbH), and the photon intensity scale was standardized using Quickview3000 software (LABATECH GmbH). The ratio of the increase in bioluminescence flux in the tumor area of each mouse between the two tests was calculated.

### TUNEL (TdT-mediated dUTP nick-end labeling) assay

The paraffin sections (*n* = 3 per group) were deparaffinized and rehydrated, followed by protease K repair and membrane permeabilization. TUNEL working solution (Servicebio, G1501) was added to the sections, which were subsequently incubated at 37 °C to label apoptotic cells. After TUNEL incubation, the sections were washed with PBS and counterstained with DAPI for nuclear visualization. The sections were then washed with PBS again, mounted with anti-fade mounting medium, and stored in the dark. Images were captured and fused using the FITC and DAPI channels of a fluorescence microscope (Carl Zeiss) at 200x magnification. Using ImageJ software, the intensity of green (FITC) fluorescence and the area of blue (DAPI) fluorescence were quantified in each immunofluorescence image. The mean green fluorescence intensity per image, which is indicative of the level of apoptosis, was subsequently calculated.

### ARS (alizarin red S) staining

Paraffin sections were deparaffinized and rehydrated with dewaxing solution and absolute ethanol, followed by staining with alizarin red solution (Servicebio, G1038) at room temperature for 5 min. The sections were then rinsed with ddH2O and dried. The samples were subsequently cleared with xylene, mounted with neutral balsam, and photographed under a microscope at 200x magnification.

### Statistical analysis

Data are expressed as the means ± standard deviations (SDs). For two independent samples, a t-test was used if a normal distribution and homogeneity of variance were assumed; otherwise, Welch’s t-test was applied. For multiple independent samples, one-way ANOVA with Šídák’s post hoc test were suitable if normality and homogeneity held; otherwise, Welch’s ANOVA with Dunnett’s post hoc test was adopted. Interaction effects were assessed using factorial ANOVA (two-way or three-way) with Šídák’s post hoc test for postanalysis. When an interaction existed, focus was placed on individual effects; otherwise, focus was placed on main effects. All the statistical analyses were conducted using GraphPad Prism 9.5.1 (GraphPad Software, LLC). The criterion for statistical significance was *P* < 0.05 (two-tailed). The experiments were performed at least three times, except for those involving nude mice.

## Electronic supplementary material

Below is the link to the electronic supplementary material.

Supplementary Material 1: Figure S1. Knockdown of ITGB3 induces G1-phase arrest and counteracts G1 progression after irradiation in osteosarcoma cells. ITGB3-knockdown (ITGB3-KD) and ITGB3-negative control (ITGB3-NC) HOS cells were subjected to either 8 Gy of irradiation (IR) or non-IR treatment, and the cell cycle distribution was monitored at various time points within 24 hours posttreatment, with n = 3 per group (A). A quantitative comparison of the percentage of cells in the G1 phase was performed across different intervention groups and time points (B). Note: At the 0-h post-IR time point, none of the groups underwent IR exposure, ensuring a consistent baseline for comparison. *P＜0.05, **P＜0.01, ***P＜0.001, ns = not significant.

Supplementary Material 2: Figure S2. Knockdown of ITGB3 upregulates osteogenic differentiation with activation of the JNK pathway. The mRNA expression of core molecules in osteogenic-related pathways (including JNK, P38, ERK, Wnt/β-catenin, BMP, Hedgehog, and NOTCH pathways) was determined using qPCR (A-G) to preliminarily screen the pathways regulated by ITGB3-knockdown (n = 3 per group). *P＜0.05, **P＜0.01, ***P＜0.001, ns = not significant.

Supplementary Material 3

Supplementary Material 4

Supplementary Material 5

Supplementary Material 6

## Data Availability

No datasets were generated or analysed during the current study.

## Abbreviations

ITGB3: Integrin subunit beta 3
RNA: Ribonucleic acid
KD: Knockdown
siRNA: small interfering RNA
JNK: c-Jun N-terminal kinase
c-JUN: c-Jun proto-oncogene
RUNX2: RUNX family transcription factor 2
OCN: Osteocalcin
OPN: Osteopontin
CSCs: Cancer stem cells
RNA-seq: RNA sequencing
IR: Irradiation
qPCR: Real-time quantitative PCR
NC: Negative control
CCK-8: Cell Counting Kit-8
mRNA: messenger RNA
MAPK: Mitogen-activated protein kinase
BMP: Bone morphogenetic protein
ERK: Extracellular signal-regulated kinase
JNKi: JNK inhibitor
p-JNK: Phosphorylated JNK
p-c-JUN: Phosphorylated c-JUN
cRGD: cyclic RGD peptide
ODM: Osteogenic differentiation medium
MEM: Minimum essential medium
IHC: Immunohistochemical
ARS: Alizarin red S
TUNEL: TdT-mediated dUTP nick-end labeling
DMEM: Dulbecco’s modified Eagle’s medium
FBS: Fetal bovine serum
SDS‒PAGE: Sodium dodecyl sulfate-polyacrylamide gel electrophoresis
PVDF: Polyvinylidene difluoride
OD: Optical density
BSA: Bovine serum albumin
IOD: Integrated optical density
DPBS: Dulbecco’s phosphate-buffered saline
PBS: Phosphate-buffered saline
SD: Standard deviation
