# Supplementary figures and images for: Enhancing radiosensitivity of osteosarcoma by ITGB3 knockdown: a mechanism linked to enhanced osteogenic differentiation status through JNK/c-JUN/RUNX2 pathway activation
Source: J Exp Clin Cancer Res. 2025 May 24;44:159. doi: 10.1186/s13046-025-03417-4 (PMC12102912; doi:10.1186/s13046-025-03417-4)

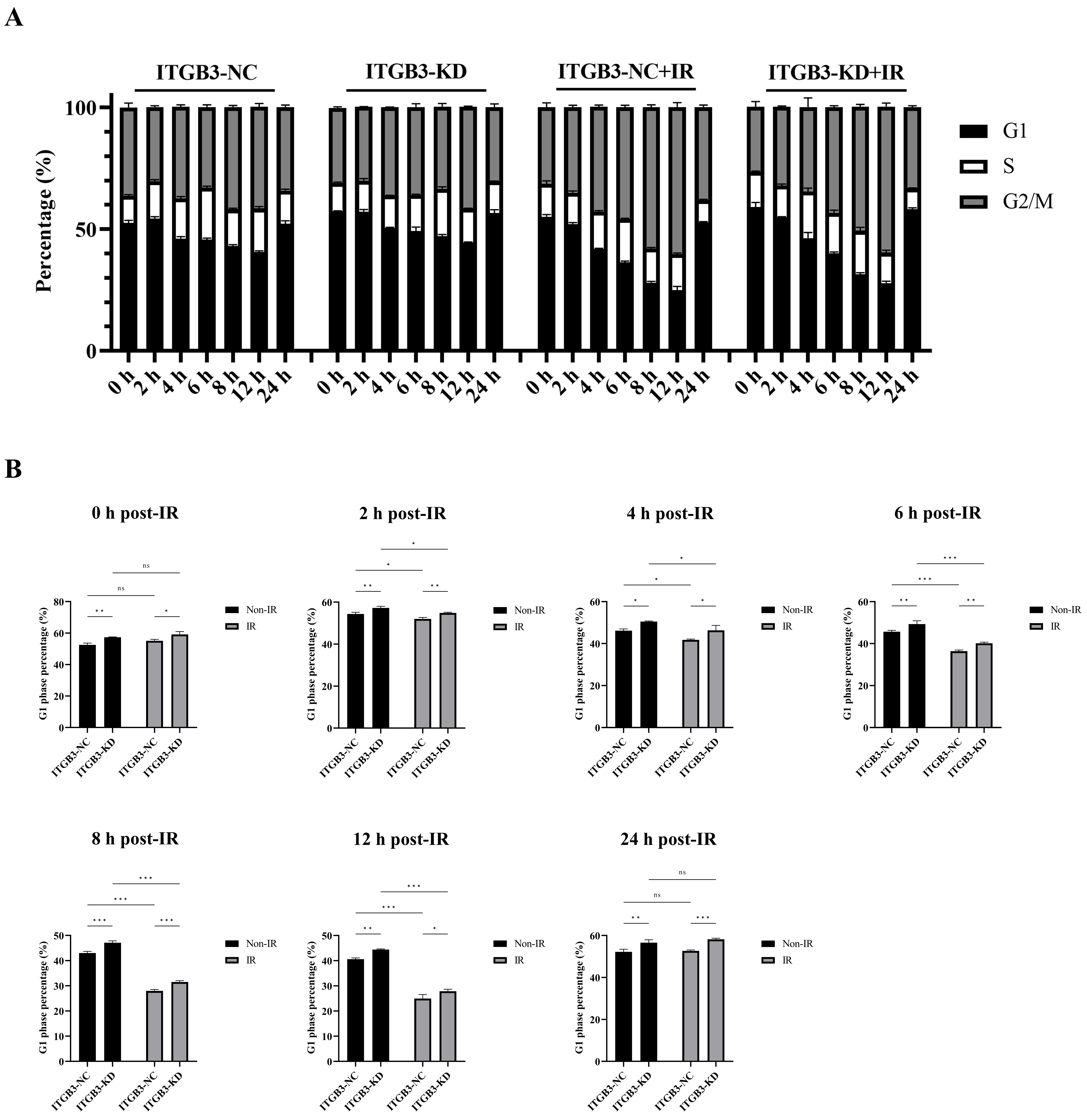

Supplement: Supplementary file 1 — Supplementary Material 1: Figure S1. Knockdown of ITGB3 induces G1-phase arrest and counteracts G1 progression after irradiation in osteosarcoma cells. ITGB3-knockdown (ITGB3-KD) and ITGB3-negative control (ITGB3-NC) HOS cells were subjected to either 8 Gy of irradiation (IR) or non-IR treatment, and the cell cycle distribution was monitored at various time points within 24 hours posttreatment, with n = 3 per group (A). A quantitative comparison of the percentage of cells in the G1 phase was performed across different intervention groups and time points (B). Note: At the 0-h post-IR time point, none of the groups underwent IR exposure, ensuring a consistent baseline for comparison. *P＜0.05, **P＜0.01, ***P＜0.001, ns = not significant. [file 13046_2025_3417_MOESM1_ESM.tif]

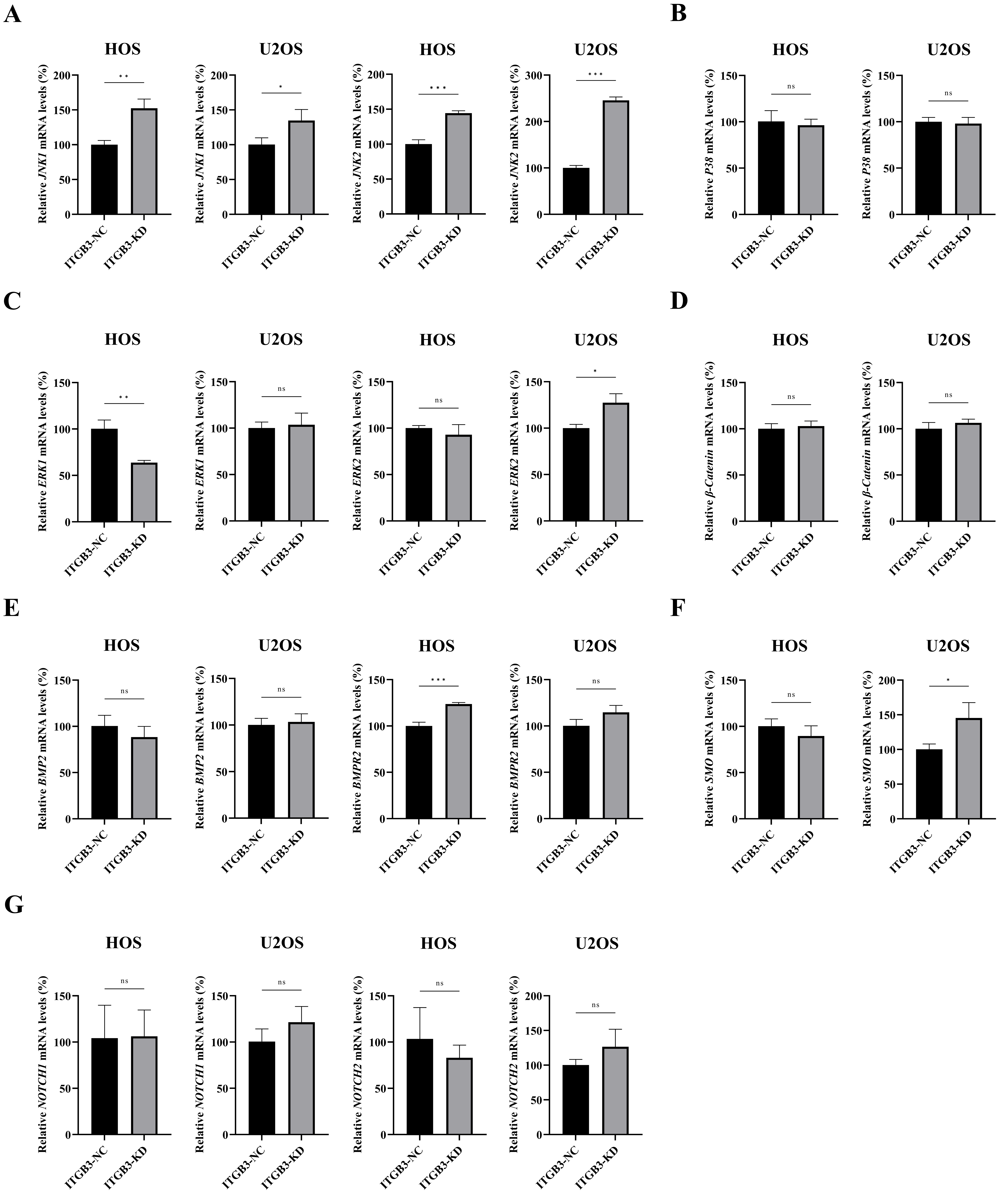

Supplement: Supplementary file 2 — Supplementary Material 2: Figure S2. Knockdown of ITGB3 upregulates osteogenic differentiation with activation of the JNK pathway. The mRNA expression of core molecules in osteogenic-related pathways (including JNK, P38, ERK, Wnt/β-catenin, BMP, Hedgehog, and NOTCH pathways) was determined using qPCR (A-G) to preliminarily screen the pathways regulated by ITGB3-knockdown (n = 3 per group). *P＜0.05, **P＜0.01, ***P＜0.001, ns = not significant. [file 13046_2025_3417_MOESM2_ESM.tif]
