# Supplementary material for: Enhancing radiosensitivity of osteosarcoma by ITGB3 knockdown: a mechanism linked to enhanced osteogenic differentiation status through JNK/c-JUN/RUNX2 pathway activation
Source: J Exp Clin Cancer Res. 2025 May 24;44:159. doi: 10.1186/s13046-025-03417-4 (PMC12102912; doi:10.1186/s13046-025-03417-4)
Supplement: Supplementary file 3 — Supplementary Material 3 [file 13046_2025_3417_MOESM3_ESM.docx]

**Table S1.** **Primers for qPCR**

| Targets | Forward primer sequence (5’ to 3’) | Reverse primer sequence (5’ to 3’) |
| --- | --- | --- |
| Human *DDX19B* | GCTTCTCCAAGGAGTCTATGCC | CAGTACCAGACTGAGATTGGGC |
| Human *CHAC1* | GTGGTGACGCTCCTTGAAGATC | GAAGGTGACCTCCTTGGTATCG |
| Human *LPP* | GTTTCACCTGCGTGATGTGCCA | GGCTGGCATAATAGGCTCCTTG |
| Human *ITGB3* | CATGGATTCCAGCAATGTCCTCC | TTGAGGCAGGTGGCATTGAAGG |
| Human *GAPDH* | GGAAGCTTGTCATCAATGGAAATC | TGATGACCCTTTTGGCTCCC |
| Human *JNK1* | GACGCCTTATGTAGTGACTCGC | TCCTGGAAAGAGGATTTTGTGGC |
| Human *JNK2* | TACGTGGTGACACGGTACTACC | CACAACCTTTCACCAGCTCTCC |
| Human *ERK1* | TGGCAAGCACTACCTGGATCAG | GCAGAGACTGTAGGTAGTTTCGG |
| Human *ERK2* | ACACCAACCTCTCGTACATCGG | TGGCAGTAGGTCTGGTGCTCAA |
| Human *P38* | GAGCGTTACCAGAACCTGTCTC | AGTAACCGCAGTTCTCTGTAGGT |
| Human *BMP2* | TGTATCGCAGGCACTCAGGTCA | CCACTCGTTTCTGGTAGTTCTTC |
| Human *BMPR2* | AGAGACCCAAGTTCCCAGAAGC | CCTTTCCTCAGCACACTGTGCA |
| Human *NOTCH1* | GGTGAACTGCTCTGAGGAGATC | GGATTGCAGTCGTCCACGTTGA |
| Human *NOTCH2* | GTGCCTATGTCCATCTGGATGG | AGACACCTGAGTGCTGGCACAA |
| Human *β-Catenin* | CACAAGCAGAGTGCTGAAGGTG | GATTCCTGAGAGTCCAAAGACAG |
| Human *SMO* | TGCTCATCGTGGGAGGCTACTT | ATCTTGCTGGCAGCCTTCTCAC |
