# Supplementary material for: Enhancing radiosensitivity of osteosarcoma by ITGB3 knockdown: a mechanism linked to enhanced osteogenic differentiation status through JNK/c-JUN/RUNX2 pathway activation
Source: J Exp Clin Cancer Res. 2025 May 24;44:159. doi: 10.1186/s13046-025-03417-4 (PMC12102912; doi:10.1186/s13046-025-03417-4)
Supplement: Supplementary file 4 — Supplementary Material 4 [file 13046_2025_3417_MOESM4_ESM.docx]

**Table S2.** **The siRNA sequence for protein expression knockdown**

| Targets | Sense strand (5’ to 3’) | Antisense strand (5’ to 3’) |
| --- | --- | --- |
| Human ITGB3 | GCUCAUCUGGAAACUCCUCAUCACC | GGUGAUGAGGAGUUUCCAGAUGAGC |
| Human RUNX2 | GAAGCUUGAUGACUCUAAA | UUUAGAGUCAUCAAGCUUC |
| Human OCN | AGCUCAAUCCGGACUGUGACGAGTT | CUCGUCACAGUCCGGAUUGAGCUTT |
| Human OPN | CCAUUCUGAUGAAUCUGAU | AUCAGAUUCAUCAGAAUGG |
