# Supplementary material for: Enhancing radiosensitivity of osteosarcoma by ITGB3 knockdown: a mechanism linked to enhanced osteogenic differentiation status through JNK/c-JUN/RUNX2 pathway activation
Source: J Exp Clin Cancer Res. 2025 May 24;44:159. doi: 10.1186/s13046-025-03417-4 (PMC12102912; doi:10.1186/s13046-025-03417-4)
Supplement: Supplementary file 5 — Supplementary Material 5 [file 13046_2025_3417_MOESM5_ESM.docx]

**Table S3.** **The lentivirus sequence for ITGB3-knockdown and ITGB3-negative control**

| Targets | Sequence (5’ to 3’) |
| --- | --- |
| Human ITGB3 | GCTCATCTGGAAACTCCTCATCACC |
| Negative control scramble | TTCTCCGAACGTGTCACGT |
