# Supplementary material for: Enhancing radiosensitivity of osteosarcoma by ITGB3 knockdown: a mechanism linked to enhanced osteogenic differentiation status through JNK/c-JUN/RUNX2 pathway activation
Source: J Exp Clin Cancer Res. 2025 May 24;44:159. doi: 10.1186/s13046-025-03417-4 (PMC12102912; doi:10.1186/s13046-025-03417-4)
Supplement: Supplementary file 6 — Supplementary Material 6 [file 13046_2025_3417_MOESM6_ESM.docx]

**Table S4.** **Antibodies for western blot and immunohistochemistry**

| Name | Company | Catalog No. |
| --- | --- | --- |
| ITGB3 Rabbit mAb | Abclonal | A19073 |
| β-Actin Rabbit mAb | Abclonal | AC026 |
| HRP-Goat Anti-Rabbit IgG(H+L) | Abclonal | AS014 |
| RUNX2 Rabbit mAb | Abclonal | A11753 |
| Osteocalcin Rabbit mAb | Abclonal | A20800 |
| Osteopontin Rabbit pAb | Affinity | AF0227 |
| JNK1/2/3 Rabbit pAb | Affinity | AF6319 |
| Phospho-JNK1/2/3(Thr183+Tyr185) Rabbit pAb | Affinity | AF3318 |
| cJUN Rabbit pAb | Abclonal | A0246 |
| Phospho-cJUN-S63 Rabbit pAb | Abclonal | AP1190 |
| BAX Rabbit mAb | Abclonal | A19684 |
| Caspase-3 Rabbit mAb | Abclonal | A25309 |
| Active + pro Caspase-3 Rabbit mAb | Abclonal | A19654 |
| Ki67 Rabbit mAb | Servicebio | GB151499 |
